# Altered ceramide metabolism is a feature in the extracellular vesicle-mediated spread of alpha-synuclein in Lewy body disorders

**DOI:** 10.1007/s00401-021-02367-3

**Published:** 2021-09-13

**Authors:** Marzena Kurzawa-Akanbi, Seshu Tammireddy, Ivo Fabrik, Lina Gliaudelytė, Mary K. Doherty, Rachel Heap, Irena Matečko-Burmann, Björn M. Burmann, Matthias Trost, John M. Lucocq, Anda V. Gherman, Graham Fairfoul, Preeti Singh, Florence Burté, Alison Green, Ian G. McKeith, Anetta Härtlova, Phillip D. Whitfield, Christopher M. Morris

**Affiliations:** 1 Biosciences Institute, International Centre for Life, Newcastle University, Central Parkway, Newcastle upon Tyne, NE1 3BZ UK; 2 Lipidomics Research Facility, Division of Biomedical Sciences, Centre for Health Science, University of the Highlands and Islands, Inverness, UK; 3 Wallenberg Centre for Molecular and Translational Medicine, University of Gothenburg, 405 30 Göteborg, Sweden; 4 Translational and Clinical Research Institute, Newcastle University, Newcastle upon Tyne, UK; 5 Department of Psychiatry and Neurochemistry, University of Gothenburg, 405 30 Göteborg, Sweden; 6 Department of Chemistry and Molecular Biology, University of Gothenburg, 405 30 Göteborg, Sweden; 7 Schools of Medicine and Biology, Medical and Biological Sciences Building, University of St Andrews, North Haugh, St Andrews, UK; 8 College of Medicine and Veterinary Medicine, The University of Edinburgh, Little France Crescent, Edinburgh, EH16 4TJ UK; 9 The National CJD Research & Surveillance Unit, Centre for Clinical Brain Sciences, University of Edinburgh, Edinburgh, EH16 4SB UK; 10 Department of Immunology and Microbiology, University of Gothenburg, 405 30 Göteborg, Sweden; 11 Present Address: Glasgow Polyomics, College of Medical, Veterinary and Life Sciences, University of Glasgow, Garscube Campus, Glasgow, G61 1QH UK

**Keywords:** Lewy body disorders, Glucocerebrosidase, Extracellular vesicles, Exosomes, Alpha-synuclein, Ceramide

## Abstract

Mutations in glucocerebrosidase (*GBA*) are the most prevalent genetic risk factor for Lewy body disorders (LBD)—collectively Parkinson’s disease, Parkinson’s disease dementia and dementia with Lewy bodies. Despite this genetic association, it remains unclear how *GBA* mutations increase susceptibility to develop LBD. We investigated relationships between LBD-specific glucocerebrosidase deficits, GBA-related pathways, and α-synuclein levels in brain tissue from LBD and controls, with and without *GBA* mutations. We show that LBD is characterised by altered sphingolipid metabolism with prominent elevation of ceramide species, regardless of *GBA* mutations. Since extracellular vesicles (EV) could be involved in LBD pathogenesis by spreading disease-linked lipids and proteins, we investigated EV derived from *post-mortem* cerebrospinal fluid (CSF) and brain tissue from *GBA* mutation carriers and non-carriers. EV purified from LBD CSF and frontal cortex were heavily loaded with ceramides and neurodegeneration-linked proteins including alpha-synuclein and tau. Our in vitro studies demonstrate that LBD EV constitute a “pathological package” capable of inducing aggregation of wild-type alpha-synuclein, mediated through a combination of alpha-synuclein–ceramide interaction and the presence of pathological forms of alpha-synuclein. Together, our findings indicate that abnormalities in ceramide metabolism are a feature of LBD, constituting a promising source of biomarkers, and that *GBA* mutations likely accelerate the pathological process occurring in sporadic LBD through endolysosomal deficiency.

**Supplementary Information:**

The online version contains supplementary material available at 10.1007/s00401-021-02367-3.

## Introduction

Lewy body disorders (LBD) encompass Parkinson’s disease (PD), Parkinson’s disease dementia (PDD) and dementia with Lewy bodies (DLB), a spectrum of disorders pathologically characterised by neuronal alpha-synuclein aggregation [1]. The end stages of PDD and DLB are clinically and neuropathologically similar suggesting that analogous pathological processes exist [43]. Glucocerebrosidase (*GBA*) gene mutations are the most prevalent genetic risk factors for LBD [65, 82], and reduced glucocerebrosidase (GBA) activity is also apparent in sporadic LBD [17, 64], suggesting a common defect in lysosomal metabolism.

GBA (EC 3.2.1.45) is a lysosomal hydrolase involved in complex glycosphingolipid catabolism, converting glucosylceramide and glucosylsphingosine into glucose and ceramide or sphingosine, respectively. Severe deficiency of GBA activity due to homozygous or compound heterozygous mutation of *GBA* is the cause of Gaucher’s disease [40]. However, accumulating evidence demonstrates that heterozygous mutation of *GBA* and subsequent reduced GBA enzymatic activity is not sufficient by itself to induce neuronal alpha-synuclein pathology, since there is a significant overlap of GBA activities between *GBA* mutation carriers and healthy controls [50]. These observations indicate that both the loss of normal function as well as a gain of toxic function by mutant GBA protein might contribute to the risk of alpha-synuclein accumulation in LBD. Although there is no evidence of significant accumulation of GBA substrates in the brains of *GBA* mutation carriers [18, 32], elevated glucosylceramide leads to the formation of high-molecular weight alpha-synuclein species [59]. Reduced GBA activity does not induce alpha-synuclein aggregation; however, it enhances pre-existing alpha-synuclein pathology [38]. The underlying mechanism behind the effects of GBA in LBD remains largely unclear; however, it potentially involves lysosomal dysfunction due to (age-related) alpha-synuclein accumulation and loss of cellular homeostasis due to GBA haploinsufficiency.

It is plausible that in *GBA* heterozygotes, lipids directly related to abnormal GBA activity are released from neurons by the exosomal transport system in a similar manner to the enhanced exosomal secretion of cholesterol in Niemann–Pick disease type C [84], which appears to be a protective mechanism to eliminate lipid accumulation in cells. Exosomes are formed via the action of endosomal sorting complexes required for transport (ESCRT) machinery or ceramide-dependent mechanisms within the endolysosomal pathway [88]. It is likely that changes in the endolysosomal system affect cargo sorting, the production of vesicles and their contents, and subsequently the inter-cellular signalling. Endoplasmic reticulum stress can induce the release of extracellular vesicles (EV) containing damage-associated molecular pattern (DAMP) molecules with a suggested role in systemic inflammation [19, 44]. This is mediated through the stress related activation of autophagy [67] in an attempt to reduce protein-related stress through the increase of secretion. Endoplasmic reticulum stress and lysosomal abnormalities are present in LBD and *GBA* mutation carriers [31, 50] and we hypothesised that EV contents might be changed in these pathologies and involved in disease pathogenesis.

The aims of the current study were to (1) provide a global lipidomic and proteomic analysis of EV from sporadic LBD and *GBA*-associated LBD post-mortem cerebrospinal fluid (CSF) and frontal cortex tissue; (2) provide brain lipid profiles from large cohorts of LBD and *GBA*-associated LBD compared to controls and elderly neuropathologically intact *GBA* controls; and (3) characterise alpha-synuclein in EV for potential aggregation-promoting properties. We demonstrate that changes in sphingolipid levels are present in LBD brain and EV, but that *GBA* carriers do not show greater sphingolipid changes. Our study also shows that neurodegeneration-linked proteins are present in LBD EV and these EV induce alpha-synuclein aggregation. These novel data add to the body of evidence that GBA modulates the risk of alpha-synucleinopathies, rather than induces it, and EV are involved in disease propagation.

## Materials and methods

### Brain tissue

Fifty-nine samples of *post-mortem* frontal cortex and 48 samples of cingulate cortex from matched LBD, LBD with heterozygous *GBA* mutations, controls with and without heterozygous *GBA* mutations were provided by the Newcastle Brain Tissue Resource. Additional samples (*n* = 2) of frontal cortex and cingulate cortex were derived from the Manchester Brain Bank (MRC ID BBN_3206 and BBN_3395) with 3 samples of frontal cortex obtained from the Edinburgh Brain and Tissue Bank (BBN_2551, BBN_2367 and BBN_2576). *Post-mortem* cerebrospinal fluid (CSF) samples (*n* = 15) were received from the Newcastle Brain Tissue Resource (Online Resource Table 1 and 2). All procedures were approved by the National Health Service Research Ethics Committee and appropriate informed consent was obtained from donors or next of kin for tissue donation.

### Purification of extracellular vesicles from *post-mortem* CSF

Frozen *post-mortem* CSF samples were thawed on ice and vortexed vigorously. CSF was pre-cleared by centrifugation: 1500×*g*, then 3000×*g* and 10,000×*g* for 10 min each at 4 °C. If more than 500 μl of CSF was available, the sample was concentrated using VivaSpin 2 centrifugal concentrators Molecular Weight Cut Off (MWCO) 3 kDa (Sartorius) to 500 μl. CSF samples, 500 µl, were then loaded onto size exclusion chromatography (SEC) columns (qEV original, Izon). Fractions were eluted with Phosphate Buffered Saline (PBS, Sigma), according to the manufacturer’s instructions. EV were contained within fractions 7, 8 and 9, 500 µl each, and those were collected to one tube and concentrated (VivaSpin 2 Sartorius MWCO 3 kDa) to 500 μl. Isolated EV were aliquoted to avoid freeze thaws and stored at − 80 °C. An aliquot of each sample was analysed for particle concentration and size by Tunable Resistive Pulse Sensing (TRPS, Izon). TRPS provides accurate measurements since it analyses individual particles as they translocate through a single nanopore compared to estimates provided by light scattering-based methods [90].

### Frozen frontal cortex extracellular vesicle purification

EV were purified from frozen frontal cortex according to an extensively modified protocol [68]. A minimum of 600 mg of frozen frontal cortex was thawed on ice and finely minced using a pair of scalpels. Tissue was then dissociated in Hibernate E (Gibco, ThermoFisher) supplemented with 5 mM L-cysteine (Sigma) and papain (Sigma, final concentration 20 units/ml) at 37 °C for 15 min with shaking. Hibernate E with protease inhibitor cocktail (Roche) was added to a total volume of 10 ml and tissue was gently homogenised by passing through a 10 ml serological pipette. Dissociated tissue was filtered using a 40 μm mesh filter (Fisher Scientific) and the filtrate centrifuged at 300×*g* for 10 min, 2000×*g* for 10 min and then 10,000×*g* for 30 min to remove cell debris and larger microvesicles. With each step, the supernatant was collected, and the final supernatant was filtered again using a 0.45 μm filter (VWR) and concentrated to 500 μl (VivaSpin Turbo 4 MWCO 3 kDa, Sartorius). This sample was applied to a SEC column (qEV original) and processed for EV isolation as previously outlined for CSF EV. Representative samples were analysed for size and concentration by means of TRPS (Izon service provision).

### Lipidomics

#### Lipid extraction

Brain tissue and EV lipids were extracted in methanol/chloroform (2/1, v/v) containing internal standards representative of major lipid classes (Online Resource Table 3). The mixture was then left to stand at 4 °C for 1 h after which it was centrifuged (750×*g* for 5 min) to remove precipitated proteins. The extract was evaporated to dryness under nitrogen gas and reconstituted in methanol containing 5 mM ammonium formate (1 ml for brain and 150 µl for EV).

#### Mass spectrometry of lipids

Lipidomic analyses were performed using a Thermo Exactive Orbitrap mass spectrometer equipped with a heated electrospray ionisation probe and coupled to a Thermo Accela 1250 UHPLC system. Brain and EV lipids were analysed in both positive and negative ion mode over the mass to charge (*m*/*z*) range 250–2000 at a resolution of 100,000. Samples were injected onto a Thermo Hypersil Gold C18 column (1.9 µm; 2.1 mm × 100 mm) maintained at 50 °C. The mobile phase A consisted of water containing 10 mM ammonium formate and 0.1% (v/v) formic acid. The mobile phase B consisted of 90:10 isopropanol/acetonitrile containing 10 mM ammonium formate and 0.1% (v/v) formic acid. The initial conditions for analysis were 65%A/35%B. The percentage of mobile phase B was increased from 35 to 65% over 4 min, followed by 65%–100% over 15 min, with a hold for 2 min before re-equilibration to the starting conditions over 6 min. The flow rate was 400 μl/min. For the targeted separation of glucosyl- and galactosylceramides in brain and EV lipid extracts, an Advanced Materials Technology HALO HILIC column (2.7 µm; 4.6 mm × 150 mm) was used according to the method of Boutin et al. 2016 [12].

#### Lipidomics data processing

The raw LC–MS data were processed with Progenesis QI software (version 2.1, Nonlinear Dynamics). Lipid identifications were made by searching against LIPID MAPS (www.lipidmaps.org/) and HMDB (http://www.hmdb.ca/).

Statistical analysis of differences in levels of quantified lipids in all groups of cases was performed by one-way ANOVA followed by a multiple comparisons test in GraphPad Prism 9.0.0.

### Proteomics

#### Protein extraction, quantification, reduction, and alkylation

EV samples were lysed in either 8 M Urea, 5 mM Triethylammonium Bicarbonate (TEAB) pH 8.5 (in solution) or 5% SDS, 50 mM TEAB pH 7.55 (S-Trap), sonicated and protein quantification was determined using the BCA Protein Assay Kit (Pierce Protein). Each sample was reduced by adding TCEP to a final concentration of 10 mM for 30 min at room temperature followed by alkylation with 10 mM iodoacetamide for 30 min in the dark.

#### S-Trap protein digestion

Samples were acidified by addition of 2.5 µl of 12% phosphoric acid and diluted with 165 µl of S-Trap binding buffer: 90% MeOH, 100 mM TEAB, pH 7.1. The acidified samples were then loaded onto an S-Trap spin column and centrifuged for 1 min at 4000×*g*. Columns were washed five times with S-Trap binding buffer before addition of porcine trypsin (1:20; Pierce) in 25 µl of 50 mM TEAB to the column. Samples were incubated at 47 °C for 2 h. Peptides were eluted by washing the column first with 50 mM TEAB, pH 8.0 (40 µl), followed by 0.2% formic acid (FA; 40 µl) and finally 0.2% FA, 50% acetonitrile (ACN; 40 µl). Peptides were then dried under vacuum before being resuspended in 0.1% TFA prior to injection.

### Mass spectrometry

#### Label-free mass spectrometry acquisition

Peptide samples were separated on an Ultimate 3000 RSLC system (Thermo Scientific) with a C18 PepMap, serving as a trapping column (2 cm × 100 µm ID, PepMap C18, 5 µm particles, 100 Å pore size) followed by a 50 cm EASY-Spray column (50 cm × 75 µm ID, PepMap C18, 2 µm particles, 100 Å pore size) (Thermo Scientific). Buffer A contained 0.1% FA and Buffer B 80% ACN, 0.1% FA. Peptides were separated with a linear gradient of 1–35% (Buffer B) over 120 min followed by a step from 35 to 90% ACN, 0.1% FA in 0.5 min at 300 nl/min and held at 90% for 4 min. The gradient was then decreased to 1% Buffer B in 0.5 min at 300 nl/min for 10 min. Mass spectrometric identification was performed on an Orbitrap QE HF mass spectrometer (Thermo Scientific) operated in “Top Speed” data dependant mode in positive ion mode. FullScan spectra were acquired in a range from 400 *m*/*z* to 1500 *m*/*z*, at a resolution of 120,000 (at 200 *m*/*z*), with an automated gain control (AGC) of 1,000,000 and a maximum injection time of 50 ms. Charge state screening was enabled to exclude precursors with a charge state of 1. For MS/MS fragmentation, the minimum AGC was set to 5000 and the most intense precursor ions were isolated with a quadrupole mass filter width of 1.6 *m*/*z* and 0.5 *m*/*z* offset. Precursors were subjected to collision induced dissociation (CID) fragmentation that was performed in one-step collision energy of 25%. MS/MS fragments ions were analysed in the Orbitrap mass analyser with a 15,000 resolution at 200 *m*/*z*.

#### Mass spectrometry data analysis

Protein identification and label-free quantification was performed using MaxQuant Version 1.6.10.43. Trypsin/P set as enzyme; stable modification carbamidomethyl (C); variable modifications Oxidation (M), Acetyl (Protein N-term), Deamidation (NQ), Gln and Glu to pyro-Glu; maximum 6 modifications per peptide, and 2 missed cleavages. Searches were conducted using the Uniprot human database (March 28, 2021) including common contaminants. Identifications were filtered at a 1% FDR at the peptide level, accepting a minimum peptide length of 5. Quantification was performed using razor and unique peptides and required a minimum count of 2. “Match between runs” was enabled. LFQ intensities were extracted for each protein/condition and used for downstream analyses. For hierarchical clustering, the intensities were first transformed to Z-score and subjected to *t* test in Perseus 1.6.2.1. Hits with *p* < 0.05 were then clustered by Euclidean distance in R using ‘pheatmap’ package and the enrichment of Gene Ontology terms in each cluster was determined by the DAVID web-based tool (https://david.ncifcrf.gov/).

### Expression and purification of alpha-synuclein

Human alpha-synuclein was expressed from plasmid pRK172 (a kind gift of Goedert) [41]. *E. coli* BL21(λ DE3) STAR cells (Novagen) were transformed with the pRK172 plasmid and grown at 37 °C in medium containing 100 µg/ml ampicillin to an OD600 ≈ 0.6 and then grown for an additional 30 min at 20 °C. Expression was induced by 1 mM IPTG. Cells were harvested 15–18 h after induction, resuspended in buffer A (25 mM Tris/HCl buffer, pH 7.7 complemented with Complete EDTA-free protease inhibitor (Roche)) at a 4:1 buffer/pellet ratio and lysed by four passes through an Emulsiflex (Avestin). The lysate was centrifuged for 30 min at 12,000×*g* at 4 °C. Purification followed a published protocol [45], briefly the supernatant was passed through a 0.22 µm filter (Merck) before being loaded onto a HiTrap Q HP column (GE Healthcare) on an Äkta Pure system (GE Healthcare). Alpha-synuclein was applied in buffer A to the column and eluted with a linear gradient to buffer A complemented with 500 mM NaCl. Alpha-synuclein containing fractions were concentrated to 2–3 ml using 10 kDa MWCO centrifugal filters (Merck). To avoid the presence of large molecular alpha-synuclein species, the protein solution was filtered through a 100 kDa MWCO centrifugal filter (Merck). The concentrated alpha-synuclein was applied to a Superdex75 column (GE Healthcare) equilibrated with PBS buffer. Fractions containing alpha-synuclein were then collected, pooled and flash frozen in liquid nitrogen before storage at -80 °C.

### Isotope labelling

We obtained [*U*-^15^N,^13^C]-labelled alpha-synuclein by growing the expression cells in M9 minimal media [76], supplemented with (^15^NH_4_)Cl and *D*-(^13^C)-glucose. All isotopes were purchased from Sigma-Aldrich.

### NMR spectroscopy

NMR measurements were performed on a Bruker AvanceIII 800 MHz spectrometer running Topspin 3.5, equipped with a cryogenically cooled TXO triple-resonance probe. All NMR experiments were performed in NMR buffer (PBS, pH 7.4) and at a temperature of 281 K. The sequence-specific backbone resonance assignment of alpha-synuclein was taken from a previous study [16]. For titration experiments 2D [^15^N,^1^H]-SOFAST-HMQC [79] experiments were recorded with inter-scan delays of 200 ms and 1024 × 128 complex points as well as CON-IPAP spectra were acquired with 1024 complex points for the 13CO dimension and 128 increments for the ^15^N-dimension [resulting from 256 IPAP (in-phase/antiphase) increments] [8, 9].

For quantitative analysis of signal intensities, the amplitudes were corrected by differences in the ^1^H-90° pulse length, the number of scans, and the dilution factor [92]. Further, a weighting function with weights 1-2-1 for residues (*i* − 1) − *i* − (*i* + 1) was applied to the raw data [15, 63]. NMR data were processed TopSpin 4.0 (Bruker BioSpin), NMRpipe [22], and mddnmr [42] as well as subsequently analysed with CARA [46]. The chemical shift changes of the amide moiety were calculated as follows:

$$\Delta \delta \left( {{\text{HN}}} \right) = \sqrt {\left( {\Delta \delta^{1} {\text{H}}} \right)^{2} + \left( {\Delta \delta^{15} {\text{N}}/5} \right)^{2} } .$$

### Real-time quaking-induced conversion (RTQuIC) assay

Protein aggregation assay was performed as described previously [23]. Fifteen μl of purified EV were subjected to RTQuIC against positive (alpha-synuclein pre-formed fibrils 1:1000 dilution of 1 mg/ml stock) and negative controls (unseeded reaction). Increase in Thioflavin T fluorescence was measured every 15 min (Ex/Em 450/480 nm) using a FLUOstar OPTIMA plate reader.

### Brain tissue homogenate preparation and fractionation

Samples of frozen cingulate cortex tissue were thawed and dissected on ice and homogenised in 5 volumes (w/v) of 0.2 M Triethylammonium bicarbonate buffer (TEAB, Sigma) supplemented with 5 mM EDTA (Sigma) and protease inhibitors cocktail (Roche) using a Precellys Evolution homogeniser. Homogenates were centrifuged at 25,830×*g* for 1 h at 4 °C and supernatants were labelled as TEAB-soluble fractions. Pellets were washed with the homogenisation buffer and resuspended in homogenisation buffer with addition of 1% Triton-X100 (final concentration, Sigma). Samples were then vortexed extensively, incubated on ice for 20–30 min and spun at 25,830×*g* for 1 h at 4 °C. Supernatant samples were labelled as Triton-X100-soluble fractions. Pellets were re-extracted in the same buffer and spun as before. Supernatants were stored but not used in the following assays and pellets were washed and resuspended in 2.5 volumes (of the original amount of homogenisation buffer) of TEAB buffer containing 8% SDS/8 M Urea. Samples were spun at 25,830×*g* for 1 h at 22 °C. Supernatants were labelled as SDS/Urea-soluble fractions. A Pierce BCA assay kit (ThermoFisher) was used to determine protein concentration of all fractions.

### Western blot

Eighteen µl of EV extracts or 15–20 μg of total brain tissue protein were subjected to Sodium Dodecyl Sulphate-Polyacrylamide Gel Electrophoresis (SDS-PAGE; NuPAGE 4–12% Bis–Tris gel, Invitrogen) according to the manufacturer’s instructions. Tetraspanins CD63 and CD81 were analysed under non-reducing conditions. Samples were heated (70 °C, 10 min) before loading onto the gel, except for SDS/Urea brain tissue fractions which were not heated. SDS-PAGE gels were transferred to Polyvinylidene Fluoride (PVDF) membranes (iblot 2, Invitrogen). Membranes were incubated with primary antibodies overnight at 4 °C, followed by washes with 1 × Tris Buffered Saline (TBS, Santa Cruz Biotechnology)-0.1% Tween (TBST) and incubation with appropriate HRP-conjugated secondary antibodies (Dako, Agilent) for an hour at room temperature. Primary antibodies were: GBA (Sigma, 1:1000), GAPDH (Santa Cruz Biotechnology, 1:2000), Alix (Cell Signalling, 1:500), SNAP25 (Sigma, 1:2000), CD63 (Abcam, 1:500), CD81 (Abcam, 1:250), Calnexin (Abcam, 1:10,000), alpha-synuclein (Abcam, 1:5000; BD Biosciences, 1:500 (SDS/Urea fraction), 1:250 (vesicular)), VDAC1/Porin (Abcam, 1:800), Synaptophysin (Abcam, 1:20,000) and TFAM (Abcam, 1:2000). Signal was developed using Pierce ECL Plus Western blotting substrate or SuperSignal West Femto Maximum Sensitivity Substrate (ThermoFisher) and detected with an Amersham Imager 600 (GE Healthcare). Bands intensities were analysed using ImageQuant software (GE Healthcare).

### Alpha-synuclein ELISA

EV samples in PBS were thawed, supplemented with Complete mini protease inhibitor cocktail tablets (Roche) and lysed with Triton-X100 (1% final concentration) on ice. The protein concentration was determined using a micro BCA protein assay kit (Pierce, ThermoFisher). Fifty μl of the lysates in duplicate were tested using a total alpha-synuclein ELISA. The ELISA plate was coated with anti-alpha-synuclein specific 10D2 antibody (amino acids 118–127 of alpha-synuclein, Analitik Jena, 1:2000) in a coating buffer (0.2 M carbonate-bicarbonate buffer, pH 9.6) in a total volume of 100 µl per well overnight at 4 °C. Wells were washed 3 times with PBS + 0.2% Tween (PBST) and the plate blocked with 1% BSA in PBST on a rocking plate for 1 h. Following a single wash with PBST, recombinant alpha-synuclein standards (Sigma-Aldrich; 0.001–40 ng/µl) or samples were prepared in PBS + 0.2% Tween, loaded in a volume of 100 µl per well and agitated on a rocking plate for 2 h. Wells were washed three times with PBST. Detection antibody against α/β synuclein (Abcam, 1:1500) in 1% BSA in PBST was added and agitated on rocking plate for 1 h, followed by three washes with PBST. Goat anti-rabbit AP-conjugate (Santa Cruz, 1:1000) in 1% BSA in PBST was incubated on a rocking plate for 1 h, followed by three washes in PBST. pNPP (*p*-nitrophenyl phosphate; Sigma-Aldrich) substrate was added (1 mg/ml in 0.05 M sodium carbonate/0.001 M MgCl_2_), and the plate incubated at 37 °C for 30 min and absorbance recorded at 412 nm. Alpha-synuclein levels were calculated based on comparison to the standard curve and normalised using the total protein concentration.

### Negative staining and transmission electron microscopy

Five μl of EV suspension in PBS was applied to glow discharged carbon-coated copper grids for 1–2 min. The grids were wicked dry by touching with a filter paper and stained with 2% uranyl acetate. Grids were dried and examined using a Philips CM 100 Compustage (FEI) Transmission Electron Microscope and digital images were collected using an AMT CCD camera (Deben). Alpha-synuclein fibrils were stained according to the same protocol and examined on a Hitachi HT7800 transmission electron microscope using an Emsis Xarosa camera with Radius software.

### Immuno-electron microscopy

EV preparations were adsorbed for 30 or 60 min to pioloform-coated EM support grids at ice temperature. Adsorbed vesicles were contrasted for electron microscopy in films of methyl cellulose containing uranyl acetate or uranyl acetate/sodium silicotungstate as detailed [5]. All procedures were performed at ice temperature unless otherwise stated.

For surface antibody labelling, grids were washed twice in PBS before incubation with 0.5% fish skin gelatine in PBS (FSG; 5 min) followed by antibody diluted in FSG (60 min). After washes in PBS, grids were incubated with rabbit anti-mouse diluted 1:250 or 1:500 in FSG (30 min; Southern Biotech), washed in PBS and then incubated with 10 nm protein A gold (30 min; BBI, Cardiff, UK). After washes in PBS and distilled water, grids were contrasted as detailed above. For labelling of permeabilised EV, the adsorbed EV were fixed in 16% paraformaldehyde in water for 10 min followed by washes in PBS and 0.1 M glycine in PBS (10 min). The EV were then permeabilised in 0.1% Triton X100 (5 min) and transferred to FSG gelatine and processed as described above. EV were imaged in a JEOL 1200EX electron microscope equipped with a GATAN Orius 200 digital camera. EV were selected for quantification using unbiased counting rule applied to a scanning band [36] and calliper diameters measured either directly on screen or estimated using stereological techniques as previously described [33, 36].

### GBA enzyme activity assay

GBA activity assay was performed as described previously [50, 86] with minor modifications. Twenty μl of fractionated brain tissue was used and 100 μl of GBA substrate (4-MUG, 4-methylumbelliferyl-β-D-glucopyranoside, Sigma) prepared at 5 mM final concentration in assay buffer (50 mM phosphate citrate buffer, pH 5.0, 0.5% sodium taurocholate, both Sigma). The reactions were carried out in 96-well black polystyrene plates (Nunc) for 2 h at 37 °C. Control reactions with a specific GBA inhibitor, conduritol beta epoxide (CBE, Sigma), were carried out in parallel. Tissue homogenates were pre-incubated with 4 mM CBE (2 mM final concentration) for 15 min at 37 °C (inhibitor controls), following which the substrate was added. Reactions were stopped with 180 μl of 1 M glycine pH 10.5. Standards of 4-methylumbelliferone (4-MU; 300–0 nM) in 1 M glycine buffer were used to generate a reference curve. Fluorescence was measured at excitation 356 nm and emission 445 nm (ThermoFisher Varioskan Lux). GBA activity was expressed as nM of 4-MU produced per mg of total protein and corrected by the amount of CBE resistant activity. Data were analysed for normality and parametric or non-parametric tests were used as appropriate to determine statistical significance, which was considered when *p* < 0.05.

## Results

### LBD *GBA* mutation carriers do not show more extensive alpha-synuclein pathology compared to sporadic LBD

*Post-mortem* brain tissue (frontal and cingulate cortex) from clinically and neuropathologically confirmed PD, PDD and DLB patients (LBD) as well as age-matched clinically and neuropathologically confirmed controls was utilised in this study (Online Resource Table 1). The presence of *GBA* mutations (L444P, IVS2 + 1, RecNciI, L105R, N370S, E326K, T369M, E388K and R262H) in 14 DLB, 3 PDD, 4 PD, and 10 controls (Online Resource Table 1 and Online Resource Table 2) was identified using whole exome sequencing and validated by Sanger sequencing and/or restriction fragment length polymorphism [47, 50]. All PD, PDD and DLB *GBA* mutation carriers presented clinical and neuropathological features typical for the disorders as diagnosed according to McKeith’s criteria for DLB [60], MDS criteria for PD [71] and neuropathological staging according to Kosaka for DLB [49], and Braak for PD [13]. Alzheimer’s disease (AD) Braak neurofibrillary tangle stage was for DLB: range 0–4, PD: 0–2, PDD: 2–4, mode (for LBD) = 3. Control *GBA* mutation carriers were free of extensive age associated neuropathological changes and presented neuropathology typical for older individuals (AD Braak neurofibrillary tangle stage range 0–3, mode = 0). AD Braak neurofibrillary tangle stage for Lewy body disorders *GBA* non-carriers was: range 0–6, mode = 1 and control non-carriers was: range 0–3, mode = 0. The average age at death for *GBA* mutation carriers was 75.3 ± 7.6 (LBD *n* = 22) and 74.1 ± 18.5 (controls *n* = 10), and for *GBA* mutation non-carriers was 72.2 ± 10.8 (LBD *n* = 17) and 74.6 ± 10.5 (controls *n* = 16) (Online Resource Table 1).

To determine any direct relationship between alpha-synuclein protein aggregation levels and corresponding levels of GBA protein and enzyme activity, cingulate cortex samples from LBD and controls with and without *GBA* mutations were fractionated using the following solubilisation agents: TEAB, Triton X100 and SDS/Urea, to isolate soluble, membrane-bound and aggregated protein fractions, respectively. GBA protein levels and enzyme activities were statistically significantly decreased in LBD *GBA* mutation carriers (Online Resource Fig. 1) and only slightly reduced in control *GBA* mutation carriers (soluble and membrane-bound fractions). There were no differences in GBA protein levels or enzyme activities in either soluble or membrane-associated fractions between LBD and controls without *GBA* mutations (Online Resource Fig. 1). These data indicate that the presence of heterozygous *GBA* mutation leads to a reduction of GBA protein and activity levels in brain tissue.

**Fig. 1:**
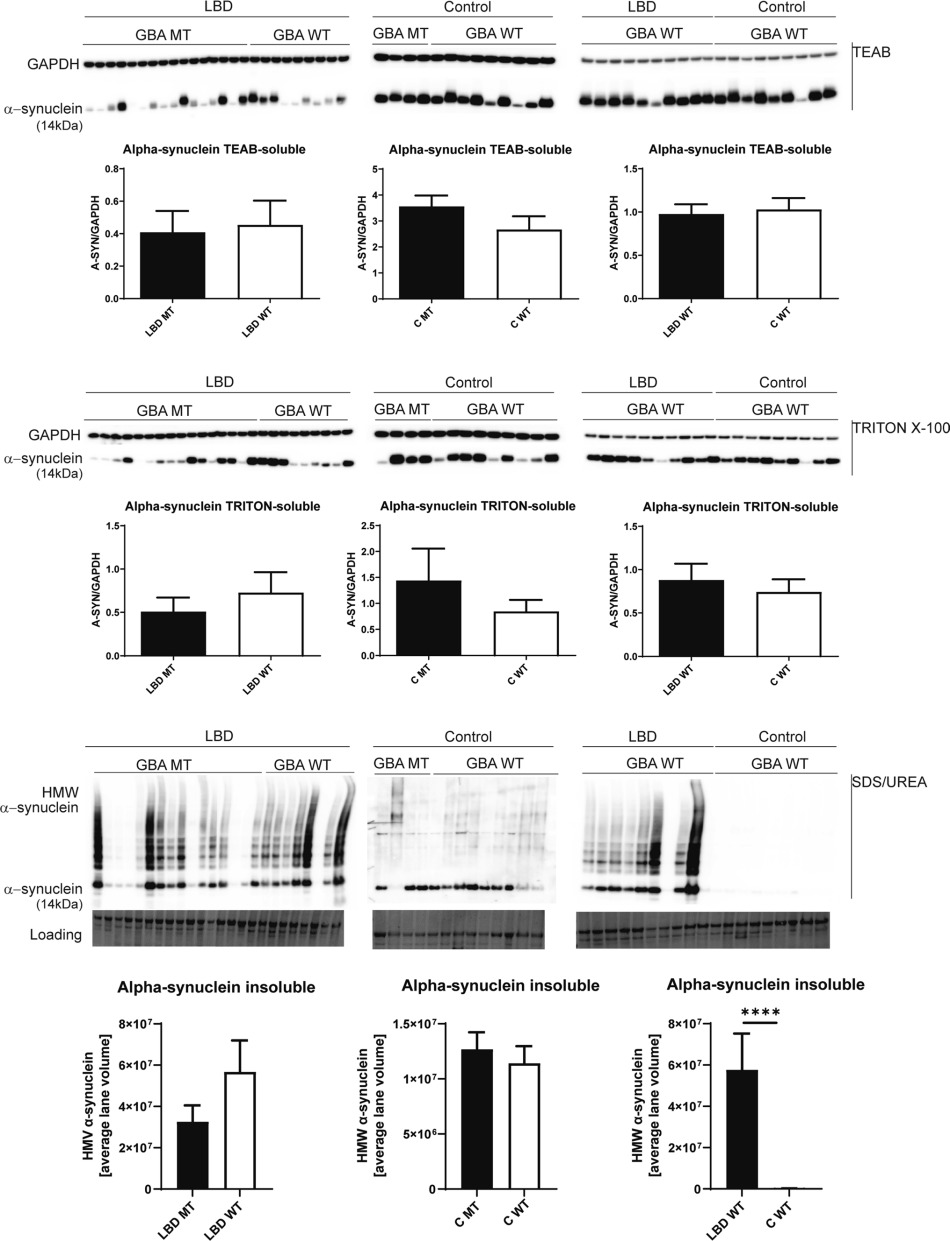
Alpha-synuclein solubility in the cingulate cortex from Lewy body disease and controls with and without *GBA* mutations. Using soluble (cytoplasmic, TEAB), detergent-soluble (membrane bound, Triton X100) and insoluble (aggregates, SDS/Urea) protein fractions from Parkinson’s disease/Dementia with Lewy body cases and controls with and without *GBA* mutations, we determined alpha-synuclein levels by western blotting. We identified the presence of high-molecular weight alpha-synuclein species only in insoluble fractions from Parkinson’s disease/Dementia with Lewy body cases with an absence in controls, but with no difference between mutation carriers (MT) and wild-type *GBA* Lewy body disorders cases (WT). Graphs represent Mean ± SEM from densitometric analysis of protein bands

Further comparative analysis revealed no significant changes in abundance of alpha-synuclein in cytoplasmic and membrane-bound protein fractions between *GBA* mutation carriers and non-carriers, LBD cases, and controls (Fig. 1). In contrast, there was an accumulation of monomeric alpha-synuclein and alpha-synuclein high-molecular weight species (aggregates) in SDS/Urea fractions in LBD patients with and without *GBA* mutations compared to controls. There was no significant difference, however, in alpha-synuclein aggregate levels between LBD with *GBA* mutations compared to LBD without *GBA* mutations (Fig. 1). Controls with *GBA* mutations did not show any extensive alpha-synuclein aggregates, which confirmed the neuropathological observations of an absence of any major alpha-synuclein pathology in controls. Overall, we observed an increase in alpha-synuclein aggregate accumulation in LBD patients, however this was independent of *GBA* mutation status.

### Ceramides are increased in the LBD brain

**Fig. 2:**
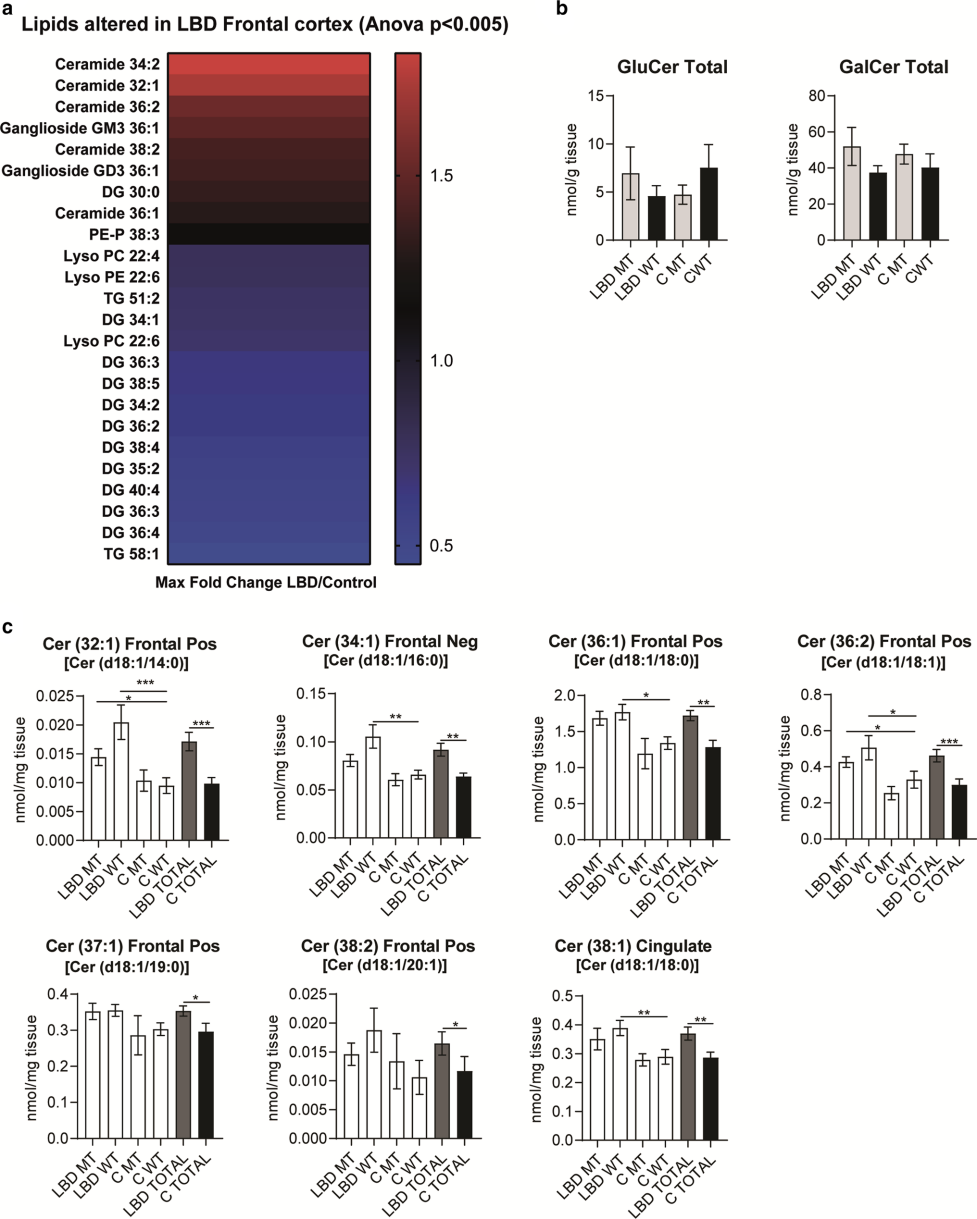
Changes in lipids between LBD cases and controls identified using global lipidomic profiling indicate altered ceramide species. **a** Initial global lipidomic profiling identified species of lipids significantly altered in LBD compared to controls within *post-mortem* frontal or cingulate cortex. The majority of changes were identified in the frontal cortex tissue. Heatmap represents maximum fold changes LBD *vs* control frontal cortex at *p* < 0.005 based on relative values from C18 column and positive ion mode. **b** No change in glucosylceramide or galactosylceramide concentrations was observed in the cingulate cortex in LBD- or control-*GBA* mutation carriers and non-carriers. Data for specific lipid species are presented in Online Resource Figs. 2 and 3. **c** Specifically focusing on ceramides, statistically significant elevations of ceramide species were identified in the frontal and cingulate cortex in the LBD group overall compared to controls with and without *GBA* mutations, with no major effect of *GBA* mutation. Ceramides have been annotated based on the combined number of carbons and double bonds of the sphingoid base and N-linked fatty acid. A putative assignment of the fatty acyl composition is also listed in parentheses. Mean ± SEM shown. Statistical analysis by one-way ANOVA followed by post hoc Dunn’s multiple comparisons test. *MT* *GBA* mutation, *WT* wild-type *GBA*, no mutation present, *TOTAL* combined groups of cases, *Pos* positive ion mode, *Neg* negative ion mode, *Cer* ceramide. The majority of changes were detected in both positive and negative ion mode, and the representative data is shown

Lipid accumulation within lysosomes is the major defect associated with GBA deficiency that causes Gaucher’s disease [14]. We, therefore, investigated potential lipid changes in relation to the overall alpha-synuclein burden (high in the cingulate versus relatively low in the frontal cortex) in LBD patients and age-matched controls. We performed a global lipidomic analysis of matched samples of *post-mortem* frontal and cingulate cortex from clinically and neuropathologically validated LBD patients and controls with and without *GBA* mutations. Hexosylceramide separation into glucosyl- and galactosylceramide species was performed on a subset of 12 LBD and 8 control cingulate cortex samples. The analysis revealed substantial differences in various lipid classes between LBD patients and controls (Fig. 2a). Notably, the most prominent changes characteristic for LBD were in the frontal cortex tissue, in contrast to subtle changes in the cingulate cortex. These included predominantly increased levels of ceramides and decreased levels of diglycerides (Fig. 2a). No changes were observed in glucosyl- and galactosylceramide species between the different groups, indicating that GBA enzymatic dysfunction is not a prerequisite for specific ceramide accumulation in LBD (Fig. 2b and Online Resource Fig. 2 and 3). We did, however, identify statistically significant increases in levels of certain ceramide species that were validated in LBD cases compared to controls using a quantitative approach, and these changes were more prevalent in the frontal cortex than the cingulate cortex (Fig. 2c). Interestingly, there were no changes characteristic of *GBA* mutation carriers in either brain region. Overall, the global lipid analysis revealed an elevation of ceramide levels in LBD patients compared to controls independent of GBA enzyme activity.

### CSF extracellular vesicles are enriched in ceramides

**Fig. 3:**
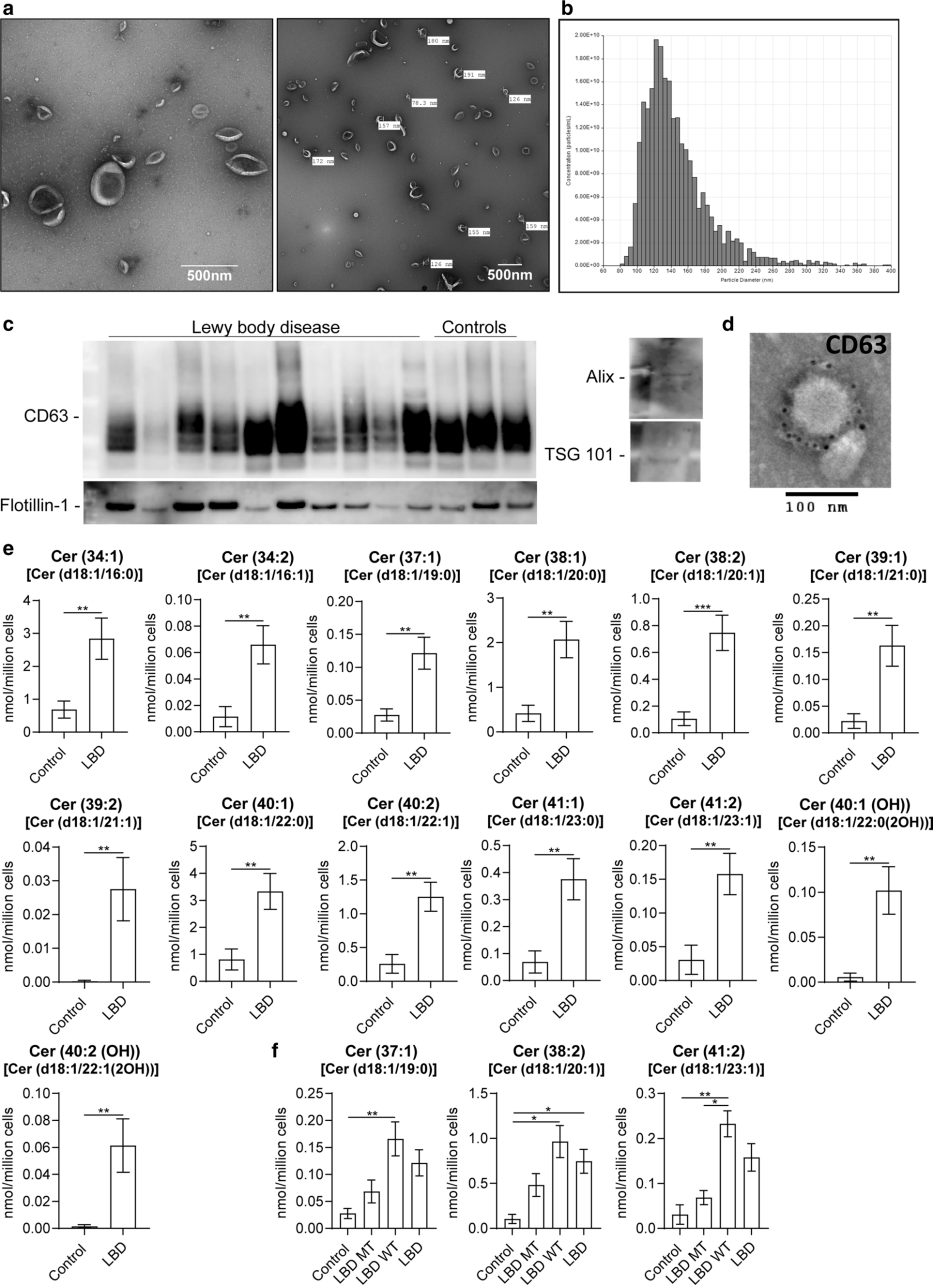
CSF extracellular vesicles are enriched in ceramides. **a** EV were purified from post-mortem CSF using size exclusion chromatography, then negatively stained and visualised using transmission electron microscopy demonstrating typical morphology. **b** EV were analysed for particle count and size by means of tunable resistive pulse sensing (TRPS) and showed a typical size of between 100 and 200 nm, with **c** western blotting demonstrating expression of known exosomal markers, and **d** CD63 immuno-labelling by immuno-electron microscopy. **e** Significantly elevated lipids, and particularly ceramides, were found in CSF extracellular vesicles from LBD compared to controls. LC–MS using C18 column in negative ion mode. Mean ± SEM shown. Statistical analysis using Mann–Whitney or unpaired *t* test with Welch’s correction. **p < 0.01, ***p < 0.001. **f** No apparent effect of *GBA* mutation was found on the levels of ceramides in LBD EV, with elevation of ceramides representing a feature of all LBD, not restricted to *GBA* mutation carriers. Representative data shown from C18 column and negative ion mode. Ceramides have been annotated based on the combined number of carbons and double bonds of the sphingoid base and N-linked fatty acid. A putative assignment of the fatty acyl composition is listed in parentheses. Mean ± SEM shown. For complete data, see Online Resource Fig. 4

We hypothesised that protein and lipid dysmetabolism in the LBD brain may influence the composition of EV that are known to have a role in inter-cellular communication and pathological alpha-synuclein distribution. We, therefore, purified EV from *post-mortem* CSF to high purity from 5 LBD (3 DLB, 1 PDD, 1 PD) cases with *GBA* mutations, 6 LBD cases (2 DLB, 2 PDD, 2 PD) without *GBA* mutations, and 4 controls without mutations in *GBA* (cases matching the brain tissue analysis). Purification of EV was performed by means of size exclusion chromatography, which preserves the morphological and functional properties of EV [11]. The yield and quality of CSF EV were determined using several methods; TRPS analysis performed on all samples determined the concentration and size of the particles (average mode for all samples 122.6 nm ± 9.9), transmission electron microscopy was used to image the particles and western blot and immuno-electron microscopy were employed to confirm the presence of known extracellular vesicles/exosome markers such as CD63, flotillin 1, Alix and Tsg101 (Fig. 3a–d). Global lipidomic analysis was performed on all EV samples. We detected statistically significant elevations in ceramide species in LBD compared to control vesicles, similar to the observations in brain tissue, with no other statistically significantly changed lipids identified and no lipids with lower levels in LBD compared to controls. Ceramides were quantified by comparison to internal standards included in the experimental system, showing a several fold increase in ceramide abundance in LBD EV compared to controls (Fig. 3e, Online Resource Fig. 5). No major effect of *GBA* mutation was seen on ceramide levels, with elevations of ceramide species being a general feature of LBD (Fig. 3f, Online Resource Fig. 4 and 6).

### Alpha-synuclein and other neurodegeneration-linked proteins are present in LBD extracellular vesicles

**Fig. 4:**
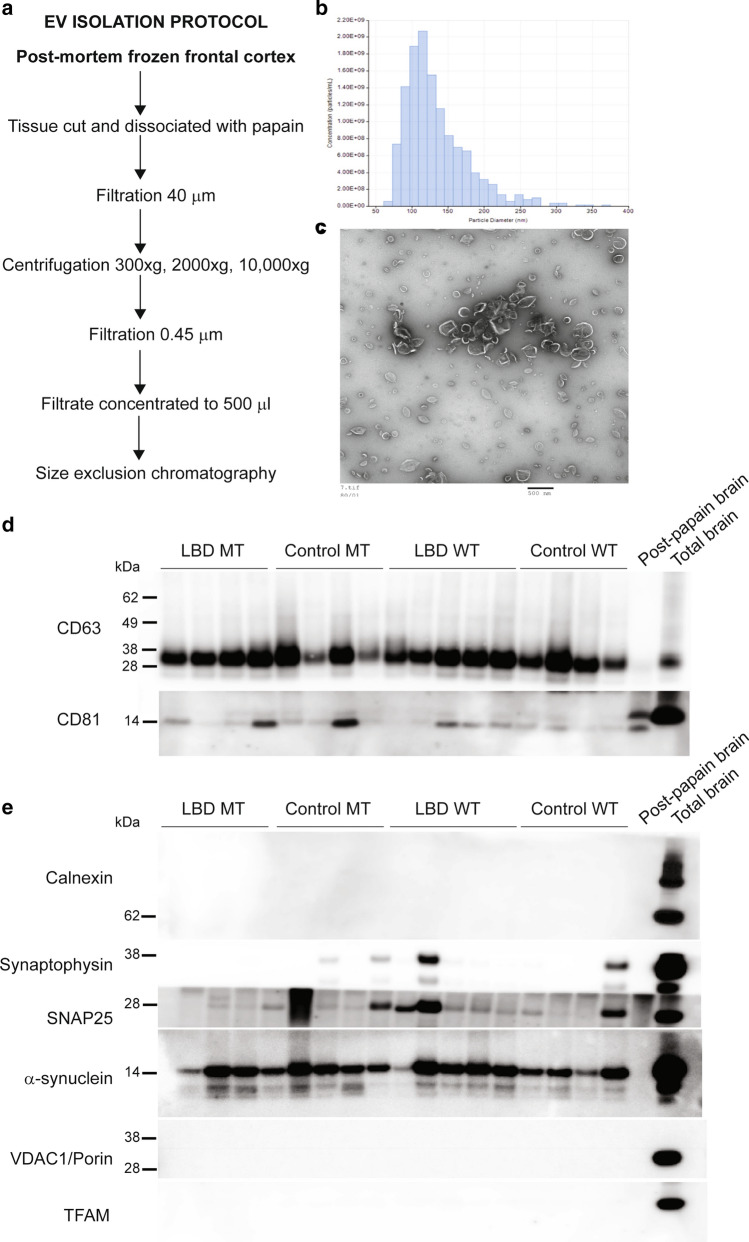
Purification of extracellular vesicles from *post-mortem* frontal cortex. **a** Frozen brain tissue was processed according to the scheme and **b** purified EV analysed for size and concentration using tunable resistive pulse sensing (TRPS) and **c** transmission electron microscopy. **d** The presence of known exosomal markers (CD63 and CD81) was verified by western blotting. **e** An absence of calnexin, VDAC1/Porin and TFAM indicates the purity of EV fractions and lack of contamination with intracellular material including mitochondria. Certain neuronal vesicle-associated proteins (synaptophysin and SNAP25) were detected in some EV preparations, although these proteins are also found in CSF EV [58]. Alpha-synuclein was present in all EV preparations from cases and controls

EV were purified from frontal cortex tissue from 13 *GBA* mutation carriers [7 LBD (5 DLB, 2 PDD) and 6 controls] and 12 *GBA* mutation non-carriers [6 LBD (3 DLB, 2 PDD, 1 PD) and 6 controls] [68] (Fig. 4a). EV were analysed for size and concentration by TRPS and imaged using transmission EM (Fig. 4b, c). The purity of EV fractions was validated based on the enrichment of exosomal markers including CD63 and CD81, while intracellular proteins such as calnexin and VDAC1/porin were absent in EV fractions, indicating the relatively high purity of the EV isolated from human brain using our approach (Fig. 4d, e). Next, we validated the presence of alpha-synuclein in all frontal cortex EV samples using western blotting (Fig. 4e), immuno-electron microscopy (Online Resource Fig. 7), and ELISA, with no differences in total alpha-synuclein levels seen between cases and controls (Online Resource Fig. 8).

**Fig. 5:**
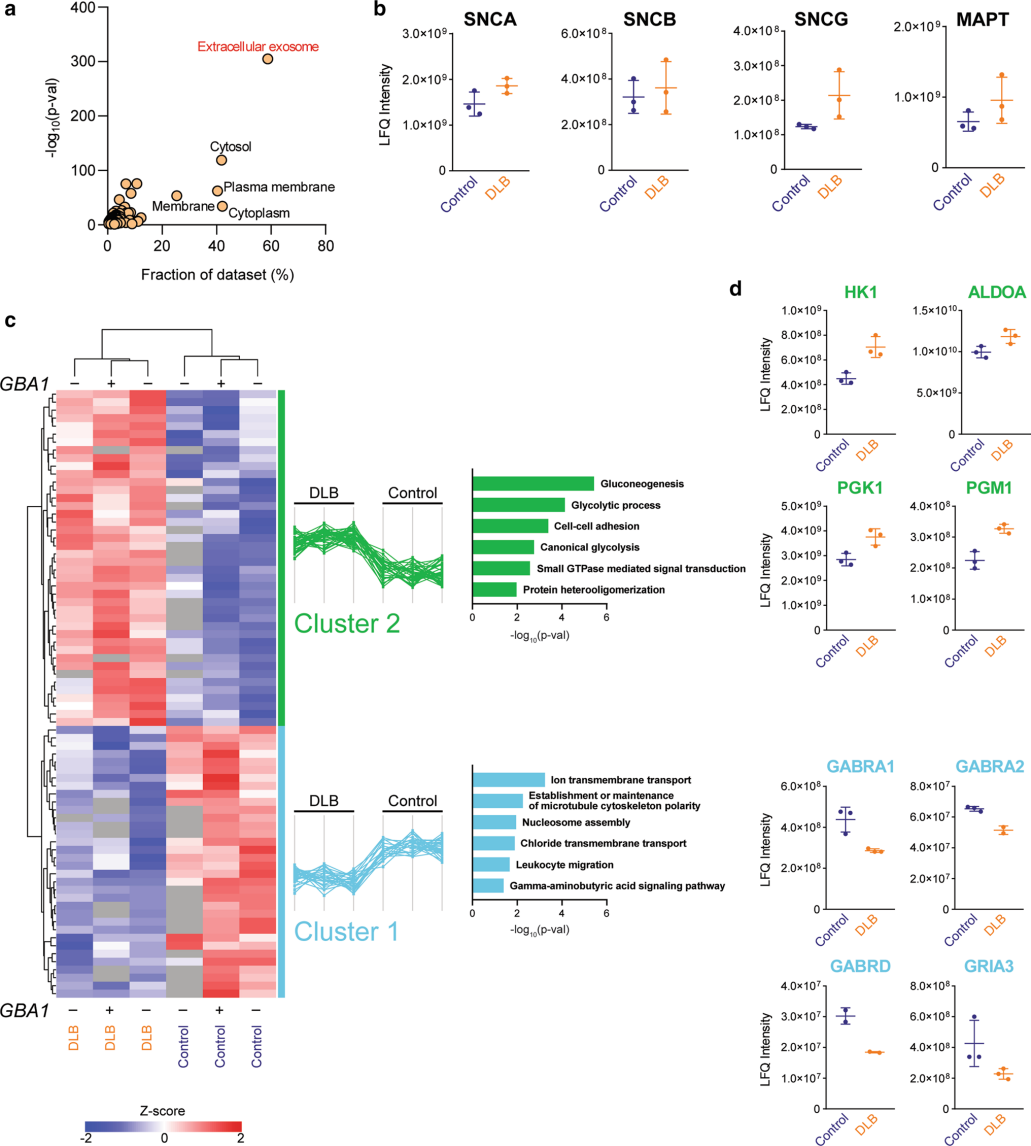
Proteomics of LBD *post-mortem* frontal cortex derived extracellular vesicles. **a** Enrichment of exosomal proteins in the frontal cortex derived EV samples. Proteins were annotated by Gene Ontology Cellular Component (GOCC) and the term enrichment was done using the DAVID web-based tool. Results are presented as a correlation of dataset fraction annotated by the given term and Benjamini–Hochberg corrected *p*-value of the enrichment test. **b** Label-free quantitation (LFQ) intensities of alpha- (SNCA), beta- (SNCB), gamma- (SNCG) synucleins and Tau protein in EV samples isolated from control and DLB patients showed the presence of proteins but no major changes in levels. **c** Hierarchical clustering of differentially expressed proteins in EV isolated from control and DLB patients. Significance was determined by *t* test. The colouring scheme corresponds to Z-score-transformed protein intensity; proteins absent in the given sample are represented by grey colour. Two clusters of proteins with similar distribution across sample types are emphasised. Gene Ontology Biological Process (GOBP) terms enrichment for each cluster was done using DAVID web-based tool and *p*-values for associated GO terms are shown in graphs of the corresponding cluster colour. Plus (+) and minus (−) denotes the presence or absence of *GBA* heterozygous mutation. **d** LFQ intensities of selected proteins annotated by cluster-specific GOBP terms. Colour of gene name corresponds to cluster membership in the heatmap

To obtain further insights into the molecular differences of disease-specific EV proteomes, we performed an exploratory label-free proteomic analysis of a subset of frontal cortex EV from DLB patients and healthy controls, with and without *GBA* mutations. This resulted in the identification of 1586 proteins (< 1% FDR) (Online Resource Table 4). Gene Ontology (GO) enrichment analysis of the frontal cortex EV proteomes confirmed the high purity of the samples (Fig. 5a). Interestingly, the proteomic analysis revealed the presence of neurodegeneration-linked proteins including alpha-synuclein, beta-synuclein, gamma-synuclein and tau protein in DLB and control EV, but with no statistically significant changes in levels (Fig. 5b, Online Resource Table 4). The presence of *GBA* mutations did not have a major effect on the EV proteome in the small sample set screened.

To identify disease-specific proteins contained within DLB frontal cortex EV, proteins with significantly altered levels were analysed by hierarchical clustering (Fig. 5c). Gene Ontology Biological Process term enrichment analysis for each differentially expressed protein cluster revealed apparent downregulation of GABAergic synaptic activity (Cluster 1; GABRA1, GABRA2, GABRD, GRIA3) and upregulation of pro-inflammatory glycolytic metabolism (Cluster2; HK1, ALDOA, PGK1, PGM1) in DLB EV (Fig. 5c, d).

**Fig. 6:**
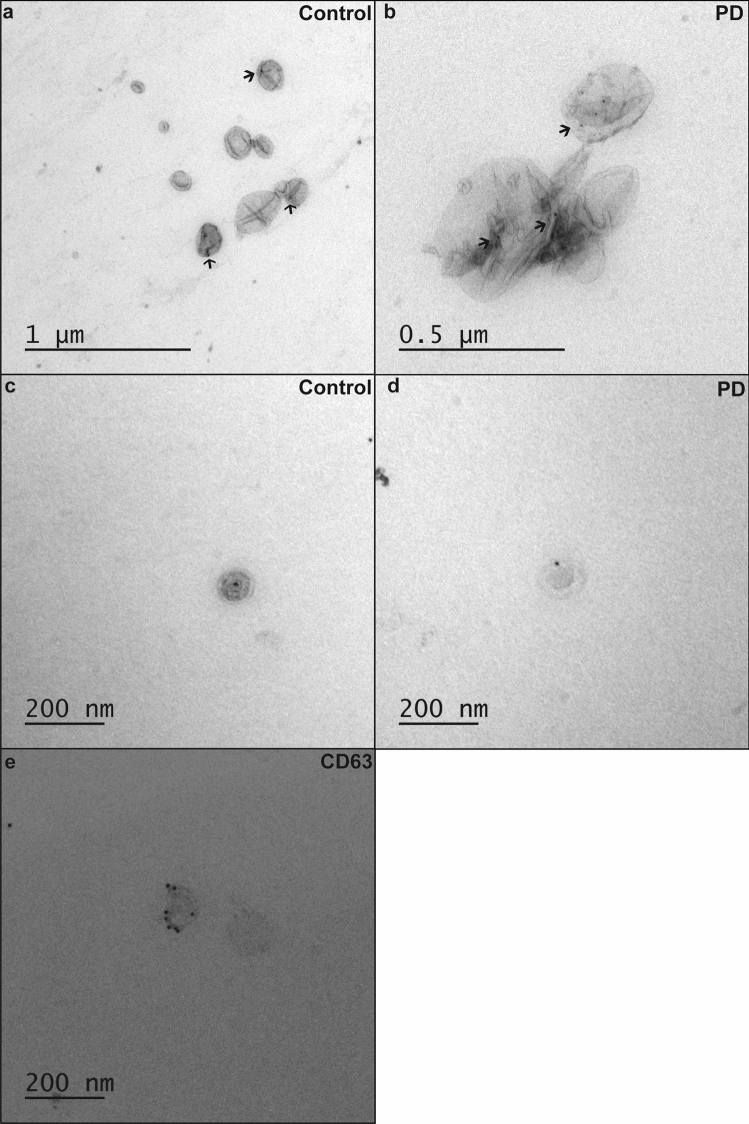
Immuno-electron microscopy of extracellular vesicles associated alpha-synuclein. **a**, **b** Alpha-synuclein was detected as membrane labelling and **c**, **d** within the vesicle interior (post-permeabilisation labelling) in CSF-derived EV from control (**a**, **c**) and LBD samples (**b**, **d**). **e** Positive CD63 labelling indicates the presence of vesicles of endosomal origin (i.e. exosomes)

Additionally, we confirmed the presence of alpha-synuclein in LBD and control *post-mortem* CSF-derived EV (surface bound and internal) using immuno-electron microscopy. We detected alpha-synuclein in both patient and control samples (9 LBD and 4 control samples screened), in association with vesicles typically 40–200 nm diameter (Fig. 6).

### Alpha-synuclein interacts with LBD EV and aggregates upon exposure

As LBD EV are filled with potentially neurotoxic lipid species and neurodegeneration-linked proteins, we thought to explore the interaction of wild-type alpha-synuclein with LBD EV. We used solution NMR spectroscopy, a technique used previously to interrogate alpha-synuclein–biological membrane binding at the atomic level [27]. Addition of equal amounts of EV samples from control and LBD frontal cortex to monomeric alpha-synuclein (Online Resource Fig. 9a), resulted in only marginal signal intensity losses without any observable chemical shift changes (e.g. resonance peak shifting) in the [^15^N,^1^H]-NMR spectra reporting on the backbone amide moiety, the standard type of experiment for studying interactions of proteins (Online Resource Fig. 9b–f). Nevertheless, exploiting the more sensitive [^15^N,^13^C]-NMR spectra [39] indicated transient interactions with disease-related EV compared to the control EV (Online Resource Fig. 9 g–k). For the different disease-related EV, an interaction within the carboxy-terminus of alpha-synuclein centred around aromatic/hydrophobic residues was observed (PDD *GBA* WT: F94, L113, Y125; PDD *GBA* MT: I112; DLB *GBA* WT: L113). The observation of effects within the carboxy-terminus of alpha-synuclein might indicate no direct vesicle interaction since this interaction occurs via the amino-terminus [57]; however, vesicle interaction of the carboxy-terminus of alpha-synuclein modulated by Ca^2+^ binding to this region enhancing its hydrophobicity has been reported [27, 52].

**Fig. 7:**
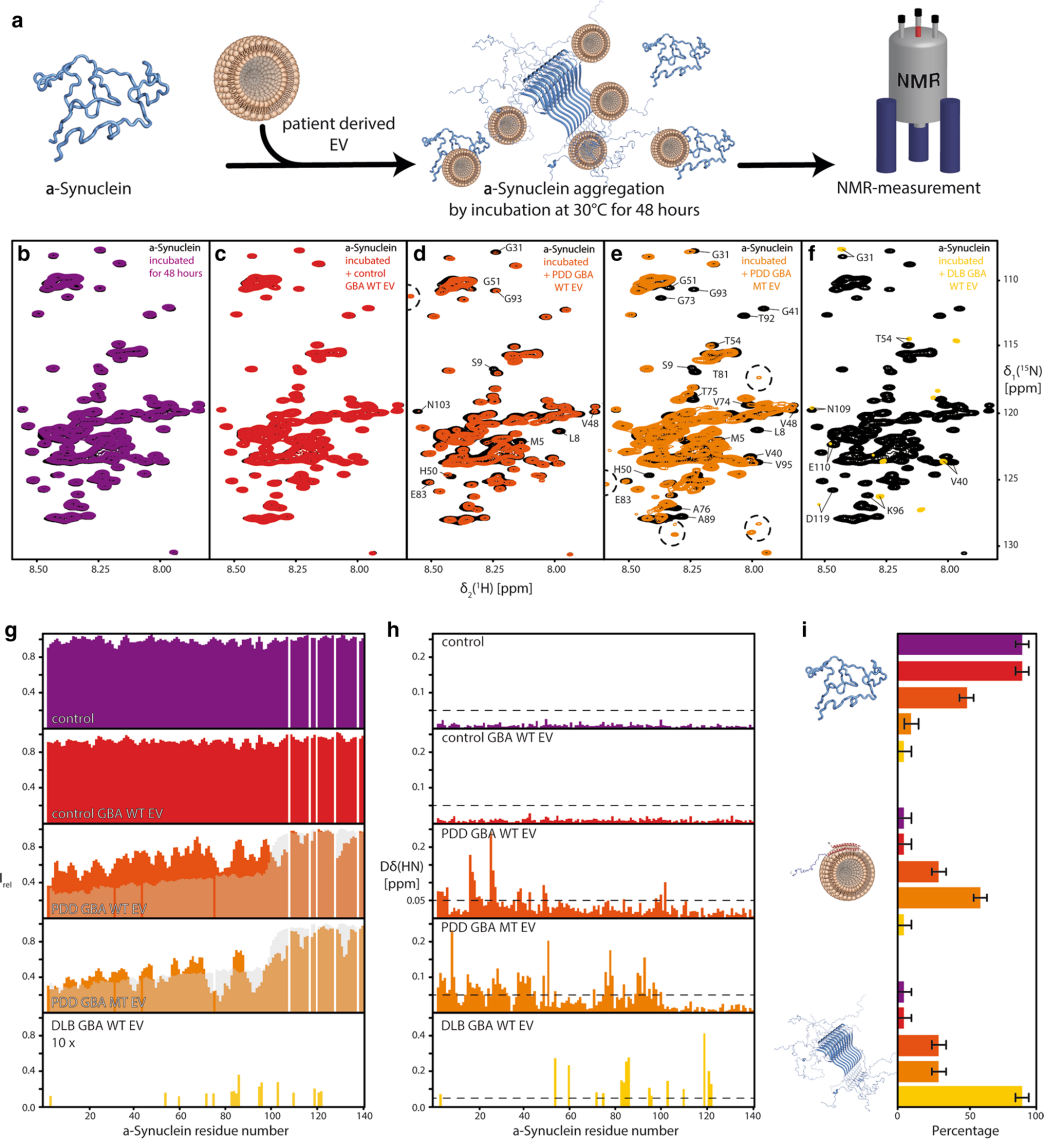
Characterisation of interactions between *post-mortem* frontal cortex derived extracellular vesicles and alpha-synuclein by NMR. **a** Scheme of the experimental set-up to investigate the effect of aggregation on the interaction between alpha-synuclein and patient-derived EV. **b**–**f** [^15^N,^1^H]-NMR spectra of 50 µM [*U*–^15^N,^13^C]-alpha-synuclein in PBS (black) and following the addition of EV of different origins as indicated after aggregation for 48 h at 30 °C. **g**, **h** Residue-resolved backbone amide NMR signal attenuation (**g**) and chemical shift changes (**h**) of alpha-synuclein upon addition of control *GBA* WT EV (red), PDD *GBA* WT EV (dark orange), PDD *GBA* MT EV (light orange), and DLB *GBA* WT EV (yellow: data multiplied by a factor of 10). As a further control, no EV were added (purple). The grey outline overlaying the PDD EV samples intensity data is alpha-synuclein in the presence of 15 mg/ml LUVs, representing the signal attenuation upon vesicle binding [16]. **i** Estimation of the distributions of monomeric alpha-synuclein, its vesicle bound and aggregated form for the tested samples

To test the effect on fibrillisation of patient-derived EV, we repeated the NMR interaction study with alpha-synuclein incubated with EV for 48 h (Fig. 7a). Under these conditions, we could not identify any effect in the absence or presence of control EV (Fig. 7b, c), whereas disease-related EV showed distinct effects (Fig. 7d–h). PDD-derived EV showed clear indications for alpha-synuclein–vesicle binding, namely signal attenuation of the amino-terminal ~ 100 amino acids similar to the effect observed for polar brain lipids [16, 87]. Furthermore, additional signals could be observed in proximity to the carboxy-terminal residues, which is a clear indication of proteolytic cleavage, which was most pronounced for the PDD *GBA* MT-derived vesicles. Overall, in both samples, a significant loss of signal intensity could be observed, indicating the presence of an NMR-indiscernible large molecular alpha-synuclein species, presumably fibrils. These aggregation-inducing effects of vesicles were particularly strong for the DLB sample analysed, which showed only very few and weak resonances (Fig. 7f). Using the three distinct species, unperturbed monomer, membrane-bound monomer indicated by the characteristic vesicle binding pattern, and the absence of NMR signal upon fibrillisation, as a proxy we estimated the contributions of these three species for the different samples tested (Fig. 7i).

The observation of the distinctive cleavage patterns in the different PDD EV prompted us to identify proteases within EV (Online Resource Fig. 10). Notably, several different cellular proteases previously implicated in alpha-synuclein processing were identified that have the potential to modulate alpha-synuclein aggregation [6, 10]. For example, the PDD *GBA* mutant sample that showed a large degree of proteolytic cleavage in the NMR spectrum, demonstrated upregulation of a subset of proteases including matrix metalloprotease ADAM22, caspase 14, and cathepsin D that can generate a C-terminal cleaved alpha-synuclein with enhanced fibrillisation propensity [10, 91]. The DLB *GBA* wild-type EV sample did not show the characteristic cleavage pattern on NMR; however, the proteomic analysis showed an upregulation of proteases mainly involved in cellular maintenance, such as subunits of the ubiquitin–proteasome system, several mitochondrial proteases (e.g. calpain 1), and oxidative stress response/alpha-synuclein chaperone activity (PARK7/DJ1) [7, 94] (Online Resource Fig. 10).

**Fig. 8:**
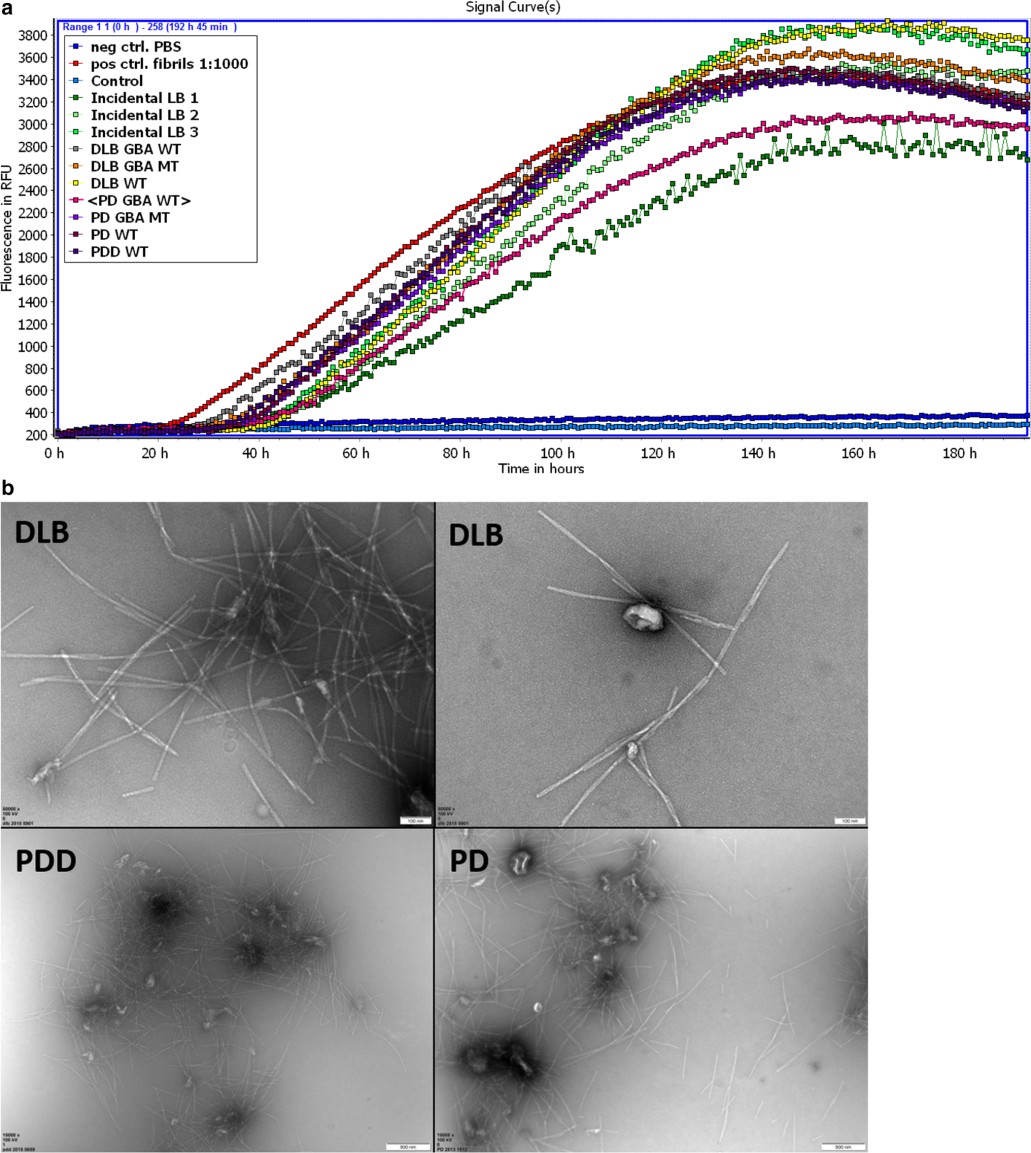
LBD patient-derived EV induce aggregation of wild-type alpha-synuclein in RTQuIC assay. **a** Exponential curves indicate the generation of alpha-synuclein aggregates upon addition of LBD-derived EV and EV derived from control individuals with incidental Lewy body pathology without LBD symptoms. Flat lines signify the lack of aggregate formation in controls (individuals with no pathology present and unseeded assay control). **b** Representative TEM images of post-RTQuIC samples from DLB (×50,000 magnification), PDD (×15,000 magnification), and PD (×15,000 magnification) individuals showing that EV are associated with the alpha-synuclein fibrils

To further validate the alpha-synuclein aggregation-inducing effect of EV, we performed real-time quaking-induced conversion (RTQuIC) assay using *post-mortem* CSF extracellular vesicles. Induction of aggregation of recombinant alpha-synuclein was observed in LBD samples, with no aggregation in controls; however, a positive signal of aggregate formation was seen in clinically normal controls with minimal alpha-synuclein pathology present (incidental Lewy bodies, Fig. 8a), indicating EV alpha-synuclein is highly fibrillogenic. TEM analysis of post-RTQuIC samples revealed abundant alpha-synuclein fibrils and on many occasions EV trapped in the fibril net, suggestive of the EV contribution to alpha-synuclein fibril generation (Fig. 8b and Online Resource Fig. 11). Overall, our findings strongly indicate that alpha-synuclein interacts with EV and aggregates upon exposure to alpha-synucleinopathy bearing EV.

## Discussion

The association between *GBA* mutations and an increased risk of developing PD and DLB is well established [65, 82], but the underlying mechanism by which GBA has its effect is still unknown. There are several potential factors that could be needed to elevate the risk of alpha-synucleinopathy, as most individuals with heterozygous *GBA* mutations do not develop LBD, with the estimated risk of developing parkinsonism at 80 years being 9.1% [4]. In the current study, we investigated the effects of heterozygous *GBA* mutations on the brain lipidome in LBD as we hypothesised that *GBA* mutations may be associated with the loss of homeostasis in lipid metabolism. We extended our hypothesis to brain secreted EV, as pathological changes in neural cells may lead to an altered composition of the EV lipidome and proteome and consequently impact disease signalling and spread to other cells.

Our study clearly indicates the specific accumulation of ceramide species in LBD signifying abnormal sphingolipid metabolism. This observation corroborates other reports of changes in sphingolipid composition of LBD *post-mortem* tissues [18, 21], though decreased ceramide levels have also been reported [2, 64, 93]. Variables such as different anatomical regions, age, mutational profiles, disease duration and/or neuropathological staging might be relevant in understanding these discrepancies [25]. Our lipidomic analysis demonstrates an elevation of ceramides irrespective of *GBA* mutation status, indicating elevated ceramides are a marker of LBD. Although ceramide levels are similar in *post-mortem GBA* mutation carriers and non-carriers, the rate of ceramide accumulation may be accelerated in *GBA* mutation carriers, and this is suggested in plasma and serum studies in LBD. Notably, increased levels of ceramides have been found in plasma of PD cases without *GBA* mutation [61], and PD *GBA* mutation carriers demonstrated elevated ceramides in serum compared to non-carriers [35]. No significant changes in CSF levels of ceramides were found between PD *GBA* mutation carriers and non-carriers; however, carriers of severe *GBA* mutations tended to have higher CSF ceramide, indicating the biochemical link between *GBA* mutations and elevated disease risk [53].

Our targeted lipidomic approach specifically analysing the levels of hexosylceramides and in particular glucosylceramides that are a substrate for GBA, indicates no change in overall levels compared to controls. This latter finding indicates that, despite mutation of GBA leading to significantly reduced enzyme activity, there is sufficient enzyme activity remaining in heterozygous mutation carriers to effectively metabolise glucosylceramides. These findings correspond with previous animal and cell work demonstrating that heterozygous mutation leads to only mild changes and retained ability to handle cellular glucosylceramide species [77]. Our results indicate that loss of GBA enzyme activity is unlikely to be responsible for the elevated ceramides observed in LBD. Therefore, we hypothesise that the elevated ceramide levels present in LBD may come from a non-lysosomal fraction.

Since EV mediate transfer of alpha-synuclein species between cells, we investigated the lipid composition of EV. Global lipidomic analysis of CSF EV revealed that LBD EV are laden with ceramide species and show markedly elevated ceramide levels when compared to control EV, indicating that the presence of elevated ceramide species is an indicator of an LBD related process. While the global lipidomic approach to ceramide species in bulk tissue samples showed modest but significant changes, the elevations of ceramides in CSF EV suggest that EV may provide a more specific indicator of ceramide dysmetabolism with changes in ceramide levels associated with EV production pathways. If ceramide dysmetabolism is an indication of underlying pathological processes associated with LBD (such as alpha-synuclein aggregation), then reductions of specific ceramide levels in CSF EV may provide an indicator of therapeutic effectiveness with agents targeting pathological processes in LBD.

We have previously shown that altered endoplasmic reticulum (ER) stress and unfolded protein response is present in LBD and in *GBA* mutation carriers [50], and suggest that the altered ceramide levels in EV may originate in the ER–Golgi system. Retention of misfolded mutant forms of GBA within the ER may underlie the higher risk and earlier age at onset of LBD, as *GBA* mutation contributes to an earlier overload of ER compared to typical late onset cases [24, 74, 75]. As the de novo synthesis of ceramide occurs in the ER [37], ceramide synthesis may be altered as a result of the loss of protein homeostasis within the ER. It has recently been shown that chemically induced ER stress in human retinal pigment epithelium cells leads to increased ceramide levels, along with increased levels of pro-apoptotic factors [3]. Alpha-synuclein accumulation can cause ER stress and inhibition of ER–Golgi trafficking, leading to cytotoxicity [20]. As ceramides are key regulators of programmed cell death [62], upregulation of ceramide may be part of a process associated with cell stress. The loss of lipid homeostasis in LBD and *GBA* mutation carriers shown here and elsewhere [18] may also indicate a general loss of lysosomal homeostasis and lysosomal degradative capacity. It has been suggested that GBA deficiency might lead to changes in the composition of lysosomal membranes, affecting alpha-synuclein degradation and other autophagic processes [80]. A pathological loop between alpha-synuclein accumulation, reduction of GBA activity through alpha-synuclein-driven blockage of lysosomal delivery, and generation of abnormal lipid profiles that might further enhance alpha-synuclein misfolding, may underlie the modifying effect of GBA in increasing susceptibility to LBD [24, 38, 59, 95].

Sphingolipids are important molecules in cellular signal transduction. Ceramide is the backbone of complex glycosphingolipids, and ceramide signalling can culminate in apoptosis, autophagy and cell cycle arrest. Cellular ceramide levels need to be balanced to regulate cell death and proliferation, a feature of a key tumour suppressor [62]. Accumulating evidence links ceramide with LBD (for review see [70]). A genetic association between PD and ASAH1, the gene coding for the acid ceramidase enzyme involved in degradation of ceramide in lysosomes has been identified [73]. Research has also shown that disruption of retromer function either by ablation of Parkinsonism related PLA2G6 and VPS35, or by overexpression of alpha-synuclein, leads to ceramide accumulation, lysosomal and mitochondrial stress, and neurodegeneration [54]. As ceramide reduction rescues the neurodegenerative phenotype, there is compelling evidence that ceramide accumulation may cause nerve cell loss [54]. Interestingly, the loss of LRRK2 (PARK8) has also been associated with retromer dysfunction and ceramide accumulation, indicating multiple genetic pathways associated with LBD involve ceramides [26, 55]. Our current study underscores the importance of the ceramide/sphingolipid pathway in sporadic LBD. The functional effect of specific ceramides may be either “physical” and involve their membrane stabilising properties and/or bioactive and related to the potent signalling functions of ceramides. Increased levels of cellular ceramides associate with decreased membrane fluidity and as a result, abnormal vesicular transport. Increased ceramides also constitute pro-apoptotic and pro-inflammatory signals, and affect mitochondrial homeostasis, common features of alpha-synucleinopathies [70]. The elevation of ceramides in LBD EV might be a mechanism of cells to dispose of excess lipids, similar to that observed in sphingolipidoses [78].

Our observations show that brain alpha-synuclein pathology is similar in LBD *GBA* mutation carriers and non-carriers. We also demonstrate that alpha-synuclein is present in EV in LBD and control samples, but with no significant changes in alpha-synuclein levels between patients and controls using ELISA and western blot analyses. It is therefore possible that an altered structure of alpha-synuclein in EV, such as fibrils, truncation, or strong alpha-synuclein–EV membrane binding detected by our NMR analysis, may be ways in which EV act as seeds for alpha-synuclein aggregation. The high abundance of heat shock 70 kDa protein (HSPA4) in DLB EV determined by mass spectrometry analysis (see Online Resource Table 4) might be an indicator of misfolded alpha-synuclein since HSPA4 can directly bind to alpha-synuclein fibrils and cause fibril disassembly [30]. Our NMR experiments indicate an initial weak binding of the C-terminus of alpha-synuclein to EV and this binding has been reported to be modulated by the presence of Ca^2+^ ions and by the presence of three distinct protein regions within alpha-synuclein [27, 52]. Using DLB EV to aggregate alpha-synuclein correlates with previous observations of alpha-synuclein membrane binding and is suggestive of a disordered C-terminus with low membrane affinity and strong membrane binding via the N-terminus of alpha-synuclein [27, 57].

The observation of the distinctive cleavage patterns in the different PDD EV by NMR correlated with the presence of proteases, such as caspases, in the EV proteomes, that have been previously implicated in facilitating alpha-synuclein aggregation in vitro [91], and likely indicating the contribution of cleaved alpha-synuclein species to the fibrillisation of these samples. In contrast, the upregulation of maintenance proteins in DLB derived EV, such as proteasomal machineries might indicate impaired proteolytic clearance possibly due to post-translational modifications of cellular alpha-synuclein impairing this process [16]. It has been shown that the impairment of the cellular protein quality control machinery leads to the accumulation of alpha-synuclein at the mitochondrial membranes [16], which may then trigger the aggregation process by stimulating primary nucleation [28]. Our observations of the upregulation of several mitochondrial proteases such as calpains (reviewed in [72]) in DLB EV may suggest impaired mitochondrial function, further supported by the upregulation of PARK7/DJ1 which is linked to oxidative stress, mitochondrial dysfunction and prevention of alpha-synuclein aggregation [94]. Impaired mitochondrial function also shows links with the recent characterisation of Lewy bodies as containing aggregated proteins and damaged organelles including mitochondria [51, 56, 81]. These observations need careful consideration however, as they were derived from a limited dataset.

Our results with EV using protein aggregation assay also strongly implicate alpha-synuclein fibrillisation upon EV membrane binding. Intact EV membranes are essential for the process of EV-mediated aggregation of alpha-synuclein [89]. It is unclear if this effect is exerted by membrane lipids, membrane curvature, membrane-associated proteins, or other factors such as those identified in this study. Nonetheless, there is accumulating evidence that vesicles are powerful inducers of alpha-synuclein fibrillisation [28, 29, 34, 85]. The altered lipid composition of LBD EV identified in this study may contribute to the abnormal alpha-synuclein misfolding and this interaction may be a major driver of the alpha-synuclein aggregation process. As ceramides are specifically enriched in LBD vesicles it may be that the abnormally high levels of ceramides are involved in the protein aggregation process in a similar manner to glucosylceramide [95] or GM1 and GM3 gangliosides, all substrates in the glycosphingolipid metabolism pathway. GM1 and GM3 gangliosides have been shown to accelerate alpha-synuclein toxic conversion in exosomes, and the role of these gangliosides in LBD warrants further validation [34]. Since we also demonstrate the presence of a wide range of neurodegeneration-linked proteins contained within LBD EV and a significant enrichment of pro-inflammatory protein signatures (see below), we suggest that EV play a role as signalosomes and transmit stress signals and disease features to other cells. EV are taken up by cells and patient-derived EV are capable of inducing alpha-synuclein aggregation in host cells [66, 85]. Our current study adds to these findings by identifying cell signalling proteins, lipid alteration and potential exchange, and cell stress stimulatory properties to the portfolio of processes that could be affected by neural EV.

Gene Ontology Biological Process term enrichment analysis of differentially expressed proteins identified in the LBD and control EV revealed apparent downregulation of GABAergic synaptic activity and upregulation of pro-inflammatory glycolytic metabolism in DLB EV. We have previously described altered GABAergic neuronal function in LBD visual cortex, and the current study indicates that functional changes in GABAergic neurons may be an inherent feature of LBD [48]. The accompanying pro-inflammatory signature of LBD EV may also reflect the activation of immune cells and the increased amounts of EV from this source in the total pool of circulating EV. Activated microglia and astrocytes switch their metabolism to glycolysis to fuel inflammation [83]. This observation correlates with elevated gene expression of microglial and astrocytic inflammatory markers in widespread cortical areas in LBD (unpublished observations) and suggest that EV may be mediators of neuroinflammation.

In summary, our findings indicate that abnormal ceramide levels are a feature of LBD independent of *GBA* mutation status, and these ceramide alterations are prominent within EV. These ceramide changes may be related to the ER stress caused by GBA protein misfolding and general loss of endolysosomal homeostasis and lysosomal degradation capacity, and consequently cause abnormal pathogenic alpha-synuclein to be associated with EV. This elevation of ceramide in CSF EV and the highly alpha-synuclein aggregation-promoting properties of EV may provide a diagnostic and therapeutic marker of LBD.

## Supplementary Information

Below is the link to the electronic supplementary material.

Supplementary file1 (DOCX 5287 KB)

Supplementary file2 (XLSX 214 KB)

## Data Availability

Data supporting this manuscript are available in the Online Resource. The mass spectrometry proteomics data have been deposited to the ProteomeXchange Consortium via the PRIDE [69] partner repository with the dataset identifier PXD026980.
