# Supplementary material for: Altered ceramide metabolism is a feature in the extracellular vesicle-mediated spread of alpha-synuclein in Lewy body disorders
Source: Acta Neuropathol. 2021 Sep 13;142(6):961–84. doi: 10.1007/s00401-021-02367-3 (PMC8568874; doi:10.1007/s00401-021-02367-3)
Supplement: Supplementary file 1 — Supplementary file1 (DOCX 5287 KB) [file 401_2021_2367_MOESM1_ESM.docx]

**Altered ceramide metabolism is a feature in the extracellular vesicle-mediated spread of alpha-synuclein in Lewy body disorders.**

Kurzawa-Akanbi et al.

**Online Resource**

**Table 1. All cases used in the study.**

| **Case ID** | **GBA mutation** | **Diagnosis** | **Age at death** | **Gender** | **AD Braak** | **McKeith staging** |
| --- | --- | --- | --- | --- | --- | --- |
| GBA MUTANT 1 | E326K | DLB | 77 | Male | 2 | Neocortical |
| GBA MUTANT 2 | N370S | DLB | 92 | Male | 4 | Neocortical |
| GBA MUTANT 3 | IVS2+1 | PDD | 73 | Female | 4 | Neocortical |
| GBA MUTANT 4 | L105R | PDD | 78 | Female | 4 | NA |
| GBA MUTANT 5 | T369M | DLB | 79 | Female | 4 | Neocortical |
| GBA MUTANT 6 | E326K | DLB | 77 | Male | 3 | Neocortical |
| GBA MUTANT 7 | N370S | DLB | 82 | Female | 3 | Neocortical |
| GBA MUTANT 8 | L444P | DLB | 81 | Male | 3 | Neocortical |
| GBA MUTANT 9 | L444P | DLB | 77 | Female | 3 | Neocortical |
| GBA MUTANT 10 | IVS2+1 | DLB | 71 | Female | 3 | NA |
| GBA MUTANT 11 | RecNciI | DLB | 57 | Female | 0 | Brain stem |
| GBA MUTANT 12 | IVS2+1 | PDD | 74 | Male | 2 | NA |
| GBA MUTANT 13 | E326K | PD | 65 | Female | 0 | Neocortical |
| GBA MUTANT 14 | L444P | DLB | 66 | Female | 2 | Neocortical |
| GBA MUTANT 15 | T369M | DLB | 72 | Female | 2 | Limbic |
| GBA MUTANT 16 | IVS2+1 | DLB | 71 | Male | 3 | Neocortical |
| GBA MUTANT 17 | L444P | PD | 69 | Male | 0 | Limbic |
| GBA MUTANT 18 | E326K | PD | 77 | Male | 2 | Neocortical |
| GBA MUTANT 19 | L444P | DLB | 79 | Male | 3 | Neocortical |
| GBA MUTANT 20 | E388K | DLB | 78 | Male | NA | NA |
| GBA MUTANT 21 | R262H | PD | 87 | Female | NA | NA |
| GBA MUTANT 22 | N370S | Control | 55 | Male | NA | NA |
| GBA MUTANT 23 | E326K | Control | 59 | Female | 0 | No LB Path |
| GBA MUTANT 24 | E326K | Control | 89 | Female | 2 | Brain stem |
| GBA MUTANT 25 | N370S | Control | 86 | Female | NA | NA |
| GBA MUTANT 26 | L444P | Control | 83 | Female | 0 | No LB Path |
| GBA MUTANT 27 | N370S | Control | 51 | Male | 3 | NA |
| GBA MUTANT 28 | T369M | Control | 96 | Female | 3 | No LB Path |
| GBA MUTANT 29 | N370S | Control | NA | NA | NA | NA |
| GBA MUTANT 30 | E326K | Control | NA | NA | NA | NA |
| GBA MUTANT 31 | N370S | Control | NA | NA | NA | NA |
| GBA WT 1 |  | DLB | 74 | Male | 4 | Neocortical |
| GBA WT 2 |  | PDD | 75 | Male | 1 | Neocortical |
| GBA WT 3 |  | DLB | 79 | Male | 2 | Neocortical |
| GBA WT 4 |  | DLB | 75 | Female | 6 | Neocortical |
| GBA WT 5 |  | DLB | 84 | Female | 6 | Neocortical |
| GBA WT 6 |  | DLB | 79 | Male | 4 | Neocortical |
| GBA WT 7 |  | DLB | 75 | Female | 6 | Diffuse |
| GBA WT 8 |  | DLB | 39 | Male | 2 | Neocortical |
| GBA WT 9 |  | DLB | 91 | Female | 5 | Limbic |
| GBA WT 10 |  | PD | 71 | Female | 2 | Neocortical |
| GBA WT 11 |  | PD | 68 | Male | 5 | Neocortical |
| GBA WT 12 |  | PD | 68 | Female | 3 | Neocortical |
| GBA WT 13 |  | PDD | 75 | Male | 5 | Neocortical |
| GBA WT 14 |  | PD | 73 | Male | 0 | Limbic |
| GBA WT 15 |  | PD | 67 | Male | 1 | Limbic |
| GBA WT 16 |  | PD | 72 | Male | 1 | Limbic |
| GBA WT 17 |  | PD | 63 | Male | 0 | Limbic |
| GBA WT 18 |  | Control | 78 | Female | 0 | No LB Path |
| GBA WT 19 |  | Control | 78 | Male | 3 | NA |
| GBA WT 20 |  | Control | 65 | Male | 0 | NA |
| GBA WT 21 |  | Control | 68 | Male | 0 | No LB Path |
| GBA WT 22 |  | Control | 103 | Female | 2 | No LB Path |
| GBA WT 23 |  | Control | 58 | Female | 0 | No LB Path |
| GBA WT 24 |  | Control | 72 | Female | 1 | No LB Path |
| GBA WT 25 |  | Control | 74 | Female | 2 | No LB Path |
| GBA WT 26 |  | Control | 83 | Male | 2 | No LB Path |
| GBA WT 27 |  | Control | 74 | Female | 1 | No LB Path |
| GBA WT 28 |  | Control | 72 | Female | 1 | NA |
| GBA WT 29 |  | Control | 63 | Male | 0 | NA |
| GBA WT 30 |  | Control | 71 | Male | 1 | Brain stem |
| GBA WT 31 |  | Control | 87 | Male | 2 | No LB Path |
| GBA WT 32 |  | Control | 77 | Male | 2 | No LB Path |
| GBA WT 33 |  | Control | 70 | Male | 0 | No LB Path |

**Table 2. *GBA* mutation cases analysed in the study.**

| ***GBA* mutation** | **DLB** | **PD/PDD** | **Controls** |
| --- | --- | --- | --- |
| **L444P** | 4 | 1 | 1 |
| **IVS2+1** | 2 | 2 | 0 |
| **RecNciI** | 1 | 0 | 0 |
| **L105R** | 0 | 1 | 0 |
| **N370S** | 2 | 0 | 5 |
| **E326K** | 2 | 2 | 3 |
| **T369M** | 2 | 0 | 1 |
| **Other** | 1 | 1 | 0 |
| **SUM** | **14** | **7** | **10** |
|  |  | **Total** | ***31*** |

**Table 3. Internal standards used for global lipidomic analysis.**

| **Item** | **ISTD** |
| --- | --- |
|  |  |
| **1** | Phosphatidylcholine (PC) 12:0/12:0 |
| **2** | Phosphatidylethanolamine (PE) 12:0/12:0 |
| **3** | Phosphatidylglycerol (PG) 14:/14:0 |
| **4** | Phosphatidylserine (PS) 14:0/14:0 |
| **5** | Phosphatidic Acid (PA) 14:0/14:0 |
| **6** | Lysophophatidylcholine (LPC) 17:0 |
| **7** | Lysophosphatidylinositol (LPI) 17:1 |
| **8** | Lysosphosphatidylethanolamione (LPE) 13:0 |
| **9** | Lysophosphatidylserine (LPS) 17:1 |
| **10** | Bis(monoacylglycero)phosphate (BMP) 14:0 |
| **11** | Dihydroceramide (DihydroCer) d18:1/12:0 |
| **12** | Ceramide (Cer) d18:1/17:0 |
| **13** | Sphingomyelin (SM) d18:1/12:0 |
| **14** | Galactosylceramide (GalCer)d18:1/12:0 |
| **15** | Lactosylceramide (LacCer) d18:1/17:0 |
| **16** | Sulfatide d18:1/12:0 |
| **17** | Cardiolipin (CL) 15:0/15:0/15:0/16:1 |
| **18** | Monoacylglycerol (MAG) 17:0 |
| **19** | Diacylglycerol (DAG) 12:0/12:0 |
| **20** | Triacylglycerol (TAG) 17:0/17:0/17:0 |
| **21** | Cholesterol Ester (ChoE) 15:0 |
| **22** | Monosialoganglioside GM1 d18:1/18:0-d3 |
| **23** | Monosialoganglioside GM2 d18:1/18:0-d3 |
| **24** | Monosialoganglioside GM3 d18:1/18:0-d3 |





**Fig. 1 GBA protein levels and enzyme activities in cingulate cortex tissue from LBD and control individuals.** Protein levels and enzyme activities were determined in fractionated cingulate cortex tissue (TEAB – soluble and Triton X-100 – membrane associated) in LBD cases and controls with and without *GBA* mutations. Mean ± SD presented, * p < 0.05, ** p < 0.01.


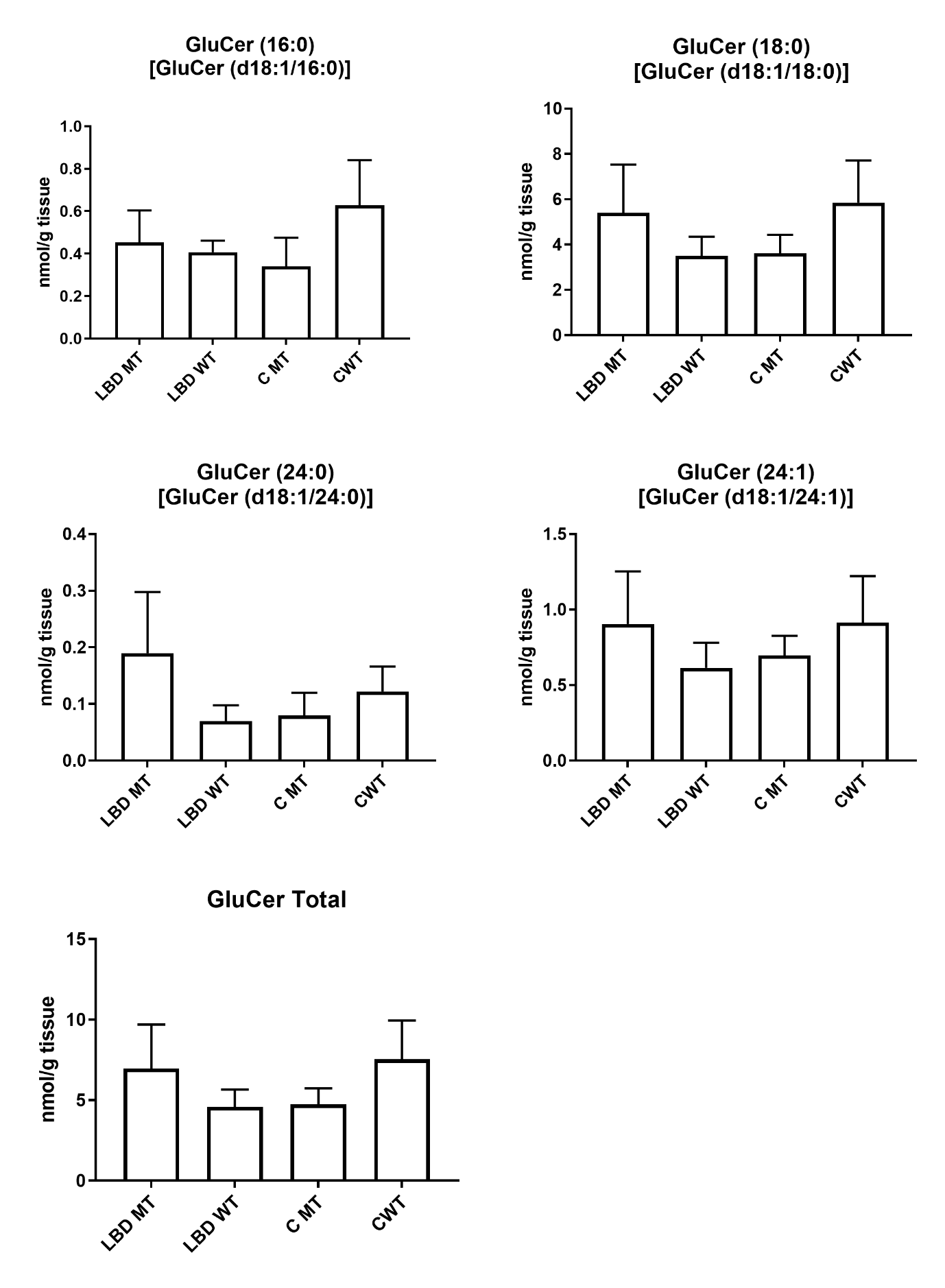
**Fig. 2 Glucosylceramide species quantification in the cingulate cortex from LBD and control *GBA* mutation carriers and non-carriers.** Individual molecular species have been annotated based on the combined number of carbons and double bonds of the sphingoid base and N-linked fatty acid. A putative assignment of the fatty acyl composition has also been listed in parentheses. No statistically significant changes in levels of lipid species were detected. Mean ± SEM presented.


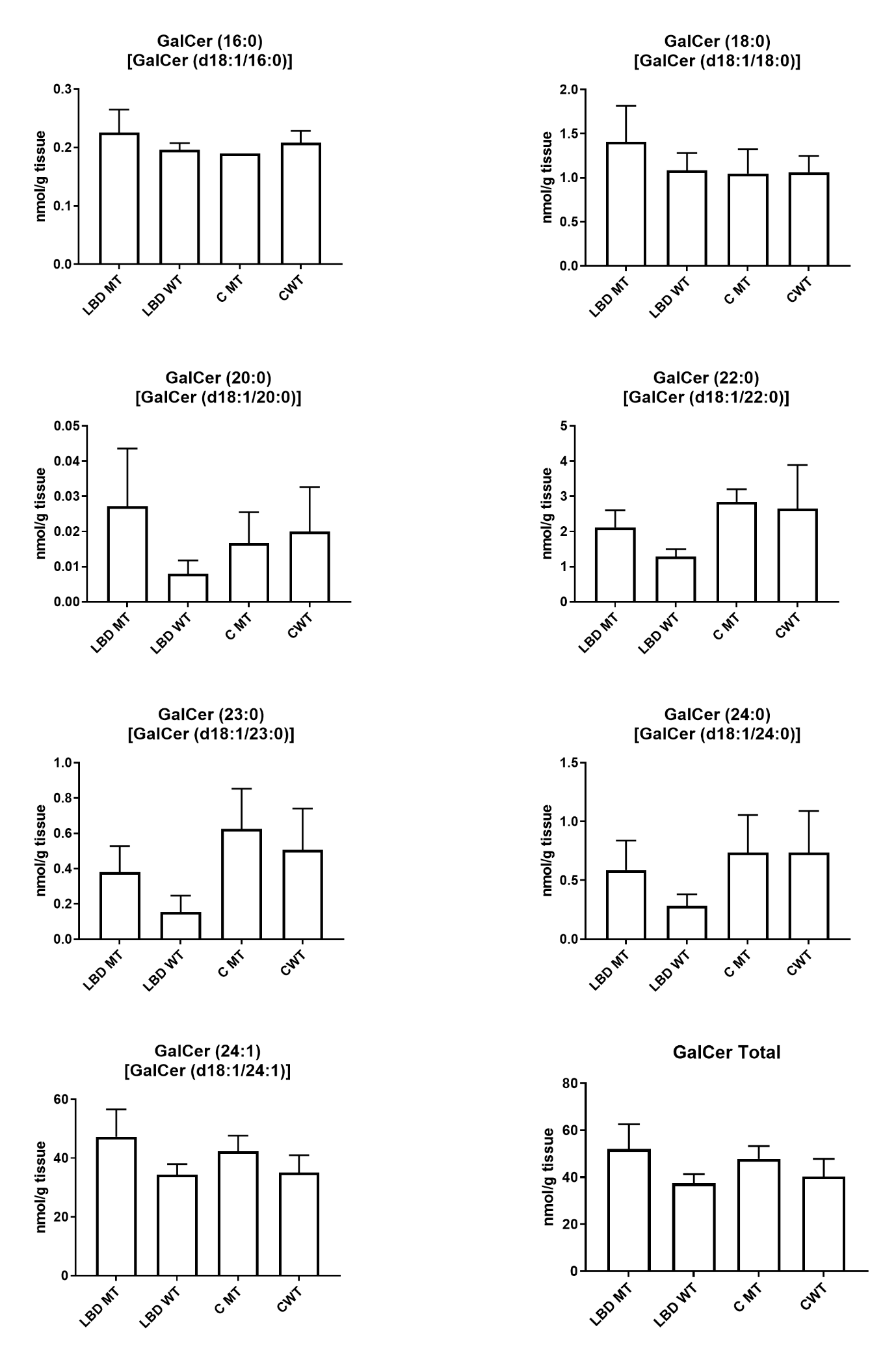


**Fig. 3 Galactosylceramide species quantification in the cingulate cortex from LBD and control *GBA* mutation carriers and non-carriers.** Individual molecular species have been annotated based on the combined number of carbons and double bonds of the sphingoid base and N-linked fatty acid. A putative assignment of the fatty acyl composition has also been listed in parentheses. No statistically significant changes in levels of lipid species were detected. Mean ± SEM presented.


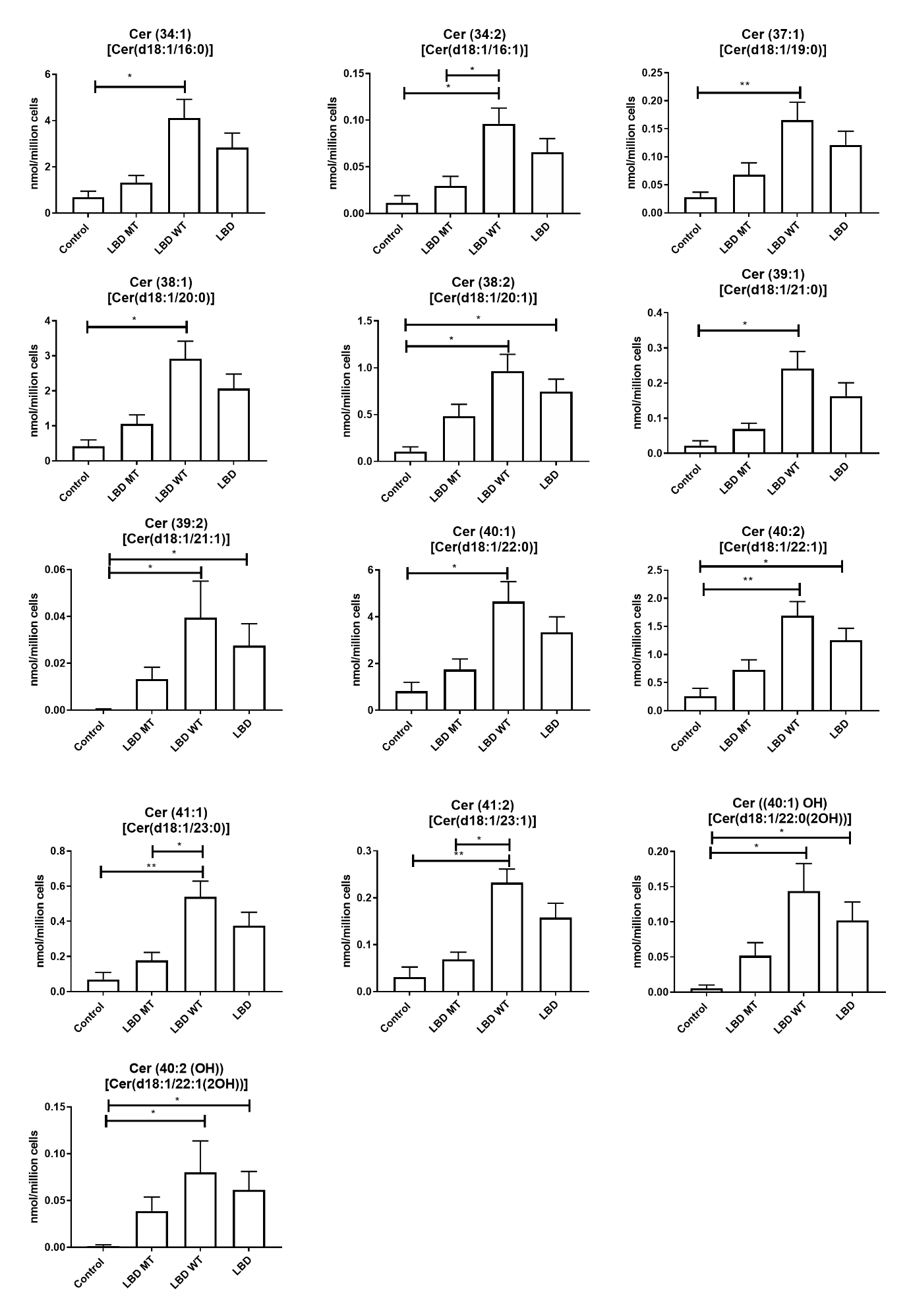


**Fig. 4 No effect of *GBA* mutation on ceramide levels in LBD extracellular vesicles (EV).** Multiple ceramide species are elevated in LBD derived EV, with elevation of ceramide representing a feature of all LBD cases that is not restricted to *GBA* mutation carriers. Data from a C18 column in negative ion mode. Ceramides have been annotated based on the combined number of carbons and double bonds of the sphingoid base and N-linked fatty acid. A putative assignment of the fatty acyl composition has also been listed in parentheses. Mean ± SEM shown. Statistical analysis by means of ANOVA with either Tukey’s or Dunn’s multiple comparisons test.


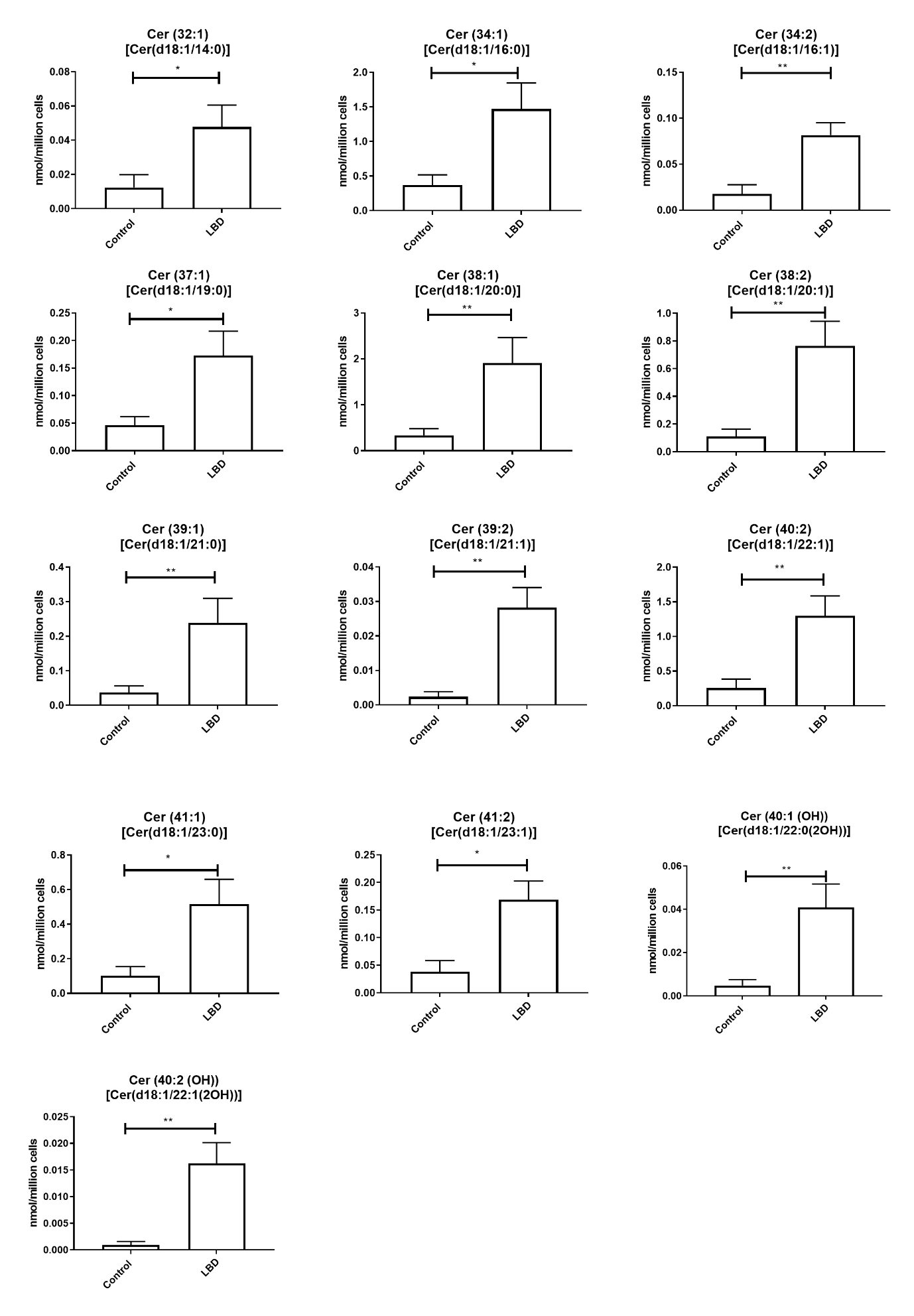


**Fig. 5 Significantly altered lipids in CSF extracellular vesicles from LBD compared to control individuals.** LC-MS using a C18 column and positive ion mode was used to determine lipid classes and their levels. Multiple ceramide species are elevated in LBD cases with Mean ± SEM presented. Statistical analysis using Mann-Whitney or unpaired t-test with Welch’s correction. * p < 0.05, ** p < 0.01. Ceramides have been annotated based on the combined number of carbons and double bonds of the sphingoid base and N-linked fatty acid. A putative assignment of the fatty acyl composition has also been listed in parentheses.


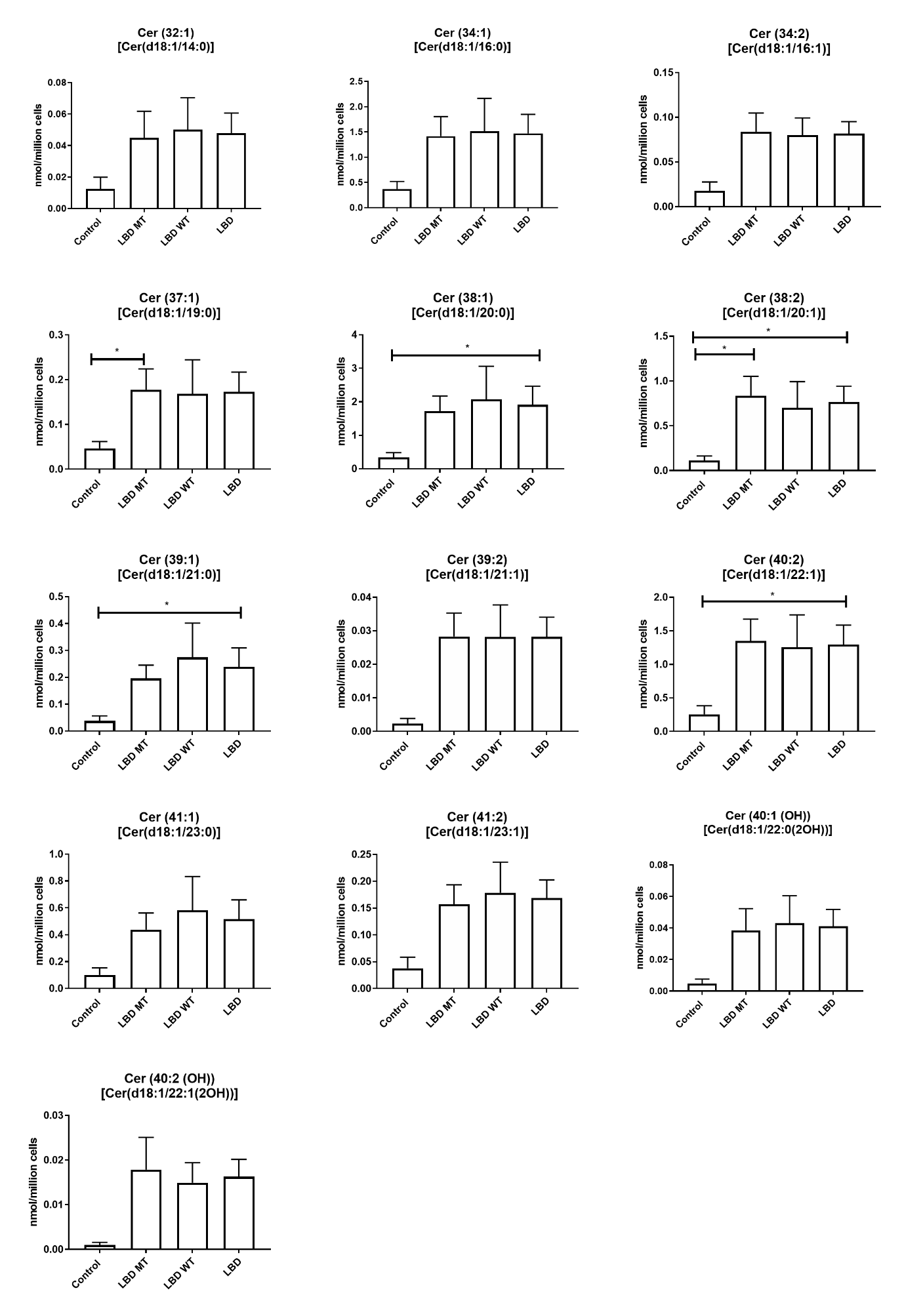


**Fig. 6 Elevated ceramide levels but no effect of *GBA* mutation in LBD EV.** Multiple ceramide species are elevated in LBD, with raised ceramide representing a general feature of all LBD cases, not restricted to *GBA* mutation carriers. Data from C18 column in positive ion mode. Ceramides have been annotated based on the combined number of carbons and double bonds of the sphingoid base and N-linked fatty acid. A putative assignment of the fatty acyl composition has also been listed in parentheses. Mean ± SEM shown. Statistical analysis by means of ANOVA with either Tukey’s or Dunn’s multiple comparisons test.


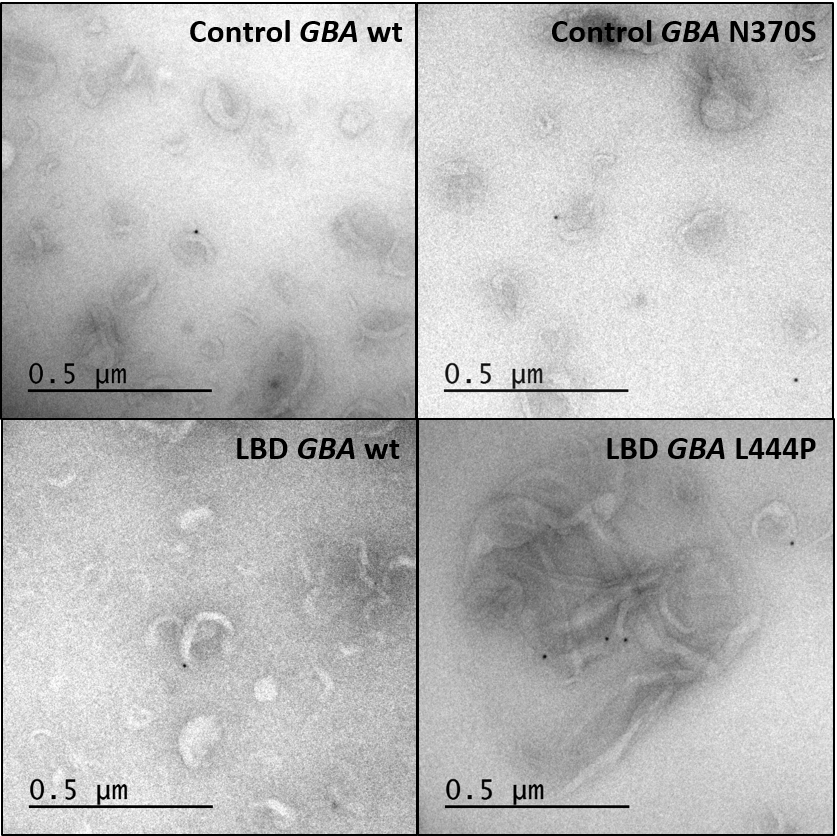


**Fig. 7 Immuno-electron microscopy of alpha-synuclein labelled frontal cortex derived vesicles.** EV were labelled with anti-alpha-synuclein antibodies and detected using gold-labelled secondary antibodies. Both small intact vesicles (e.g. control *GBA* wild type upper left panel) and disrupted vesicles (e.g. LBD *GBA* L444P) showed the presence of alpha-synuclein.

**Fig. 8 Alpha-synuclein ELISA and detection of total alpha-synuclein abundance in frontal cortex EV.** Using a sandwich ELISA (monoclonal 10D2 capture antibody, recombinant alpha-synuclein standards, α/β synuclein detection antibody) we determined levels of total alpha-synuclein in EV fractions from control and LBD cases. No significant changes in alpha-synuclein levels were observed between case and control EV. LBD MT, C MT – Lewy body disorders and controls with *GBA* mutations; LBD WT, C WT - Lewy body disorders and controls without *GBA* mutations; LBD TOTAL, C TOTAL – Lewy body disorders and controls all cases included. Mean ± SEM shown.


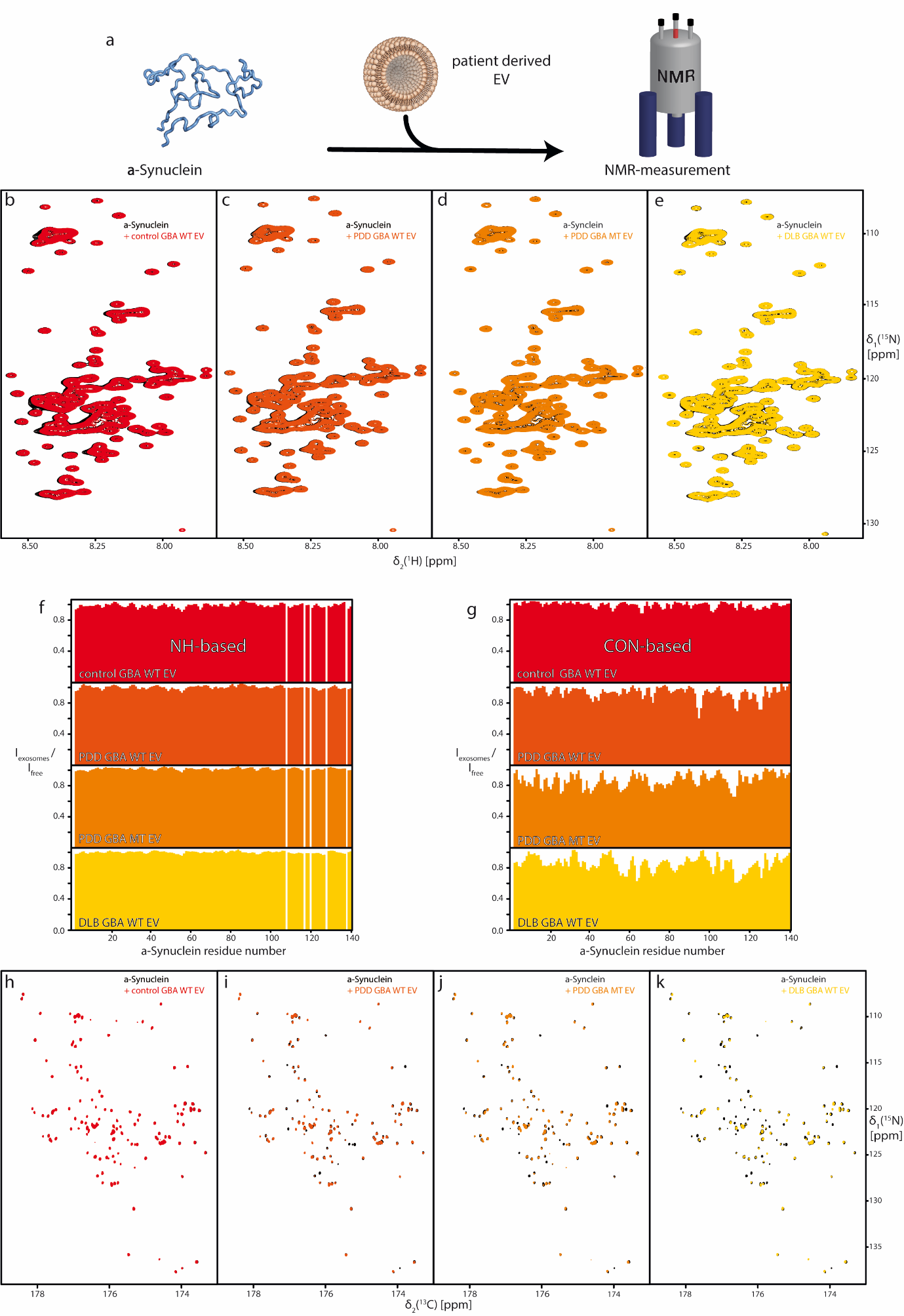


Fig. 9 Characterization of interactions between *post-mortem* frontal cortex derived extracellular vesicles and alpha-synuclein by NMR. (a) Scheme of the experimental set-up to investigate the interaction between monomeric alpha-synuclein and patient derived EV. (b–e) [^15^N,^1^H]-NMR spectra of 50 µM [*U*–^15^N,^13^C]-alpha-synuclein in PBS (black) and after addition of EV of different origin as indicated. (f, g) Residue-resolved backbone amide (NH-based) (f) and carbonyl (CON-based) (g) NMR signal attenuation of alpha-synuclein upon addition of case-derived control GBA WT EV (red), PDD GBA WT EV (dark-orange), PDD GBA MT EV (orange), and DLB GBA WT EV (yellow). (h–k) [^15^N,^13^C]-NMR spectra of 50 µM [*U*–^15^N,^13^C]-alpha-synuclein in PBS (black) and after addition of EV of different origin as indicated. All NMR spectra were measured at 10°C.


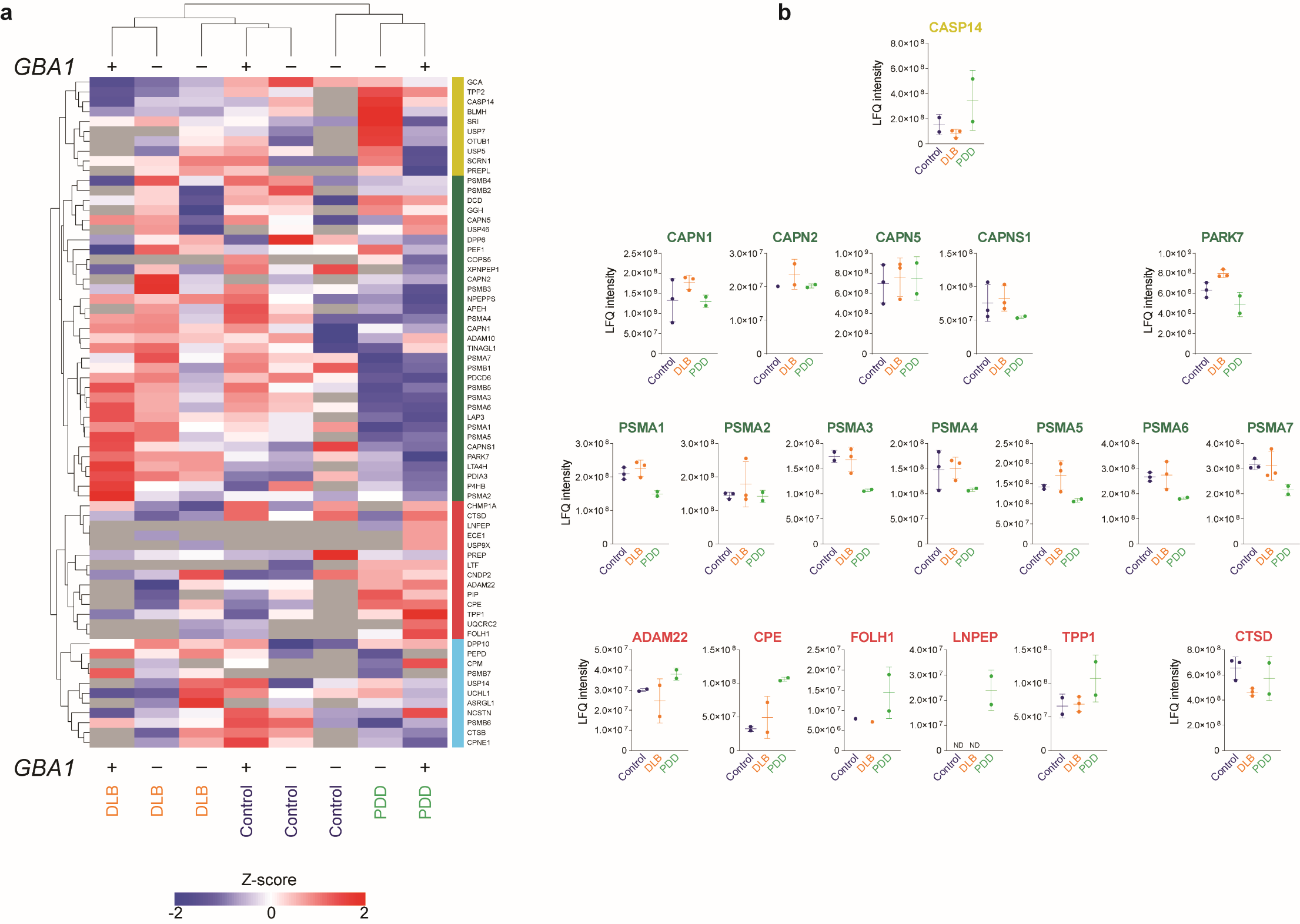


Fig. 10 Proteases identified in *post-mortem* frontal cortex derived extracellular vesicles. (a) Two PDD EV samples were processed and analyzed by LC-MS as described in material and methods and acquired spectra were searched together with data acquired for DLB and control EV samples (see Figure 5) in separate MaxQuant run using identical parameters. Identified proteins annotated by Gene Ontology Molecular Function term “peptidase activity” were clustered by hierarchical clustering. Coloring corresponds to Z-score-transformed protein intensity; proteins not detected in the given sample are represented by grey color. (b) LFQ intensities of selected proteins in EV samples. Color of gene name corresponds to cluster membership in heatmap. CASP14 – caspase 14; CAPN – calpain, CAPNS1 – calpain small subunit 1; PARK7 – protein deglycase DJ-1; PSMA – proteasome subunit alpha; ADAM22 – disintegrin and metalloproteinase domain-containing protein 22; metalloproteases: CPE – carboxypeptidase E, FOLH1 – glutamate carboxypeptidase 2, LNPEP – leucyl-cystinyl aminopeptidase, TPP1 – tripeptidyl-peptidase 1; CTSD – cathepsin D.


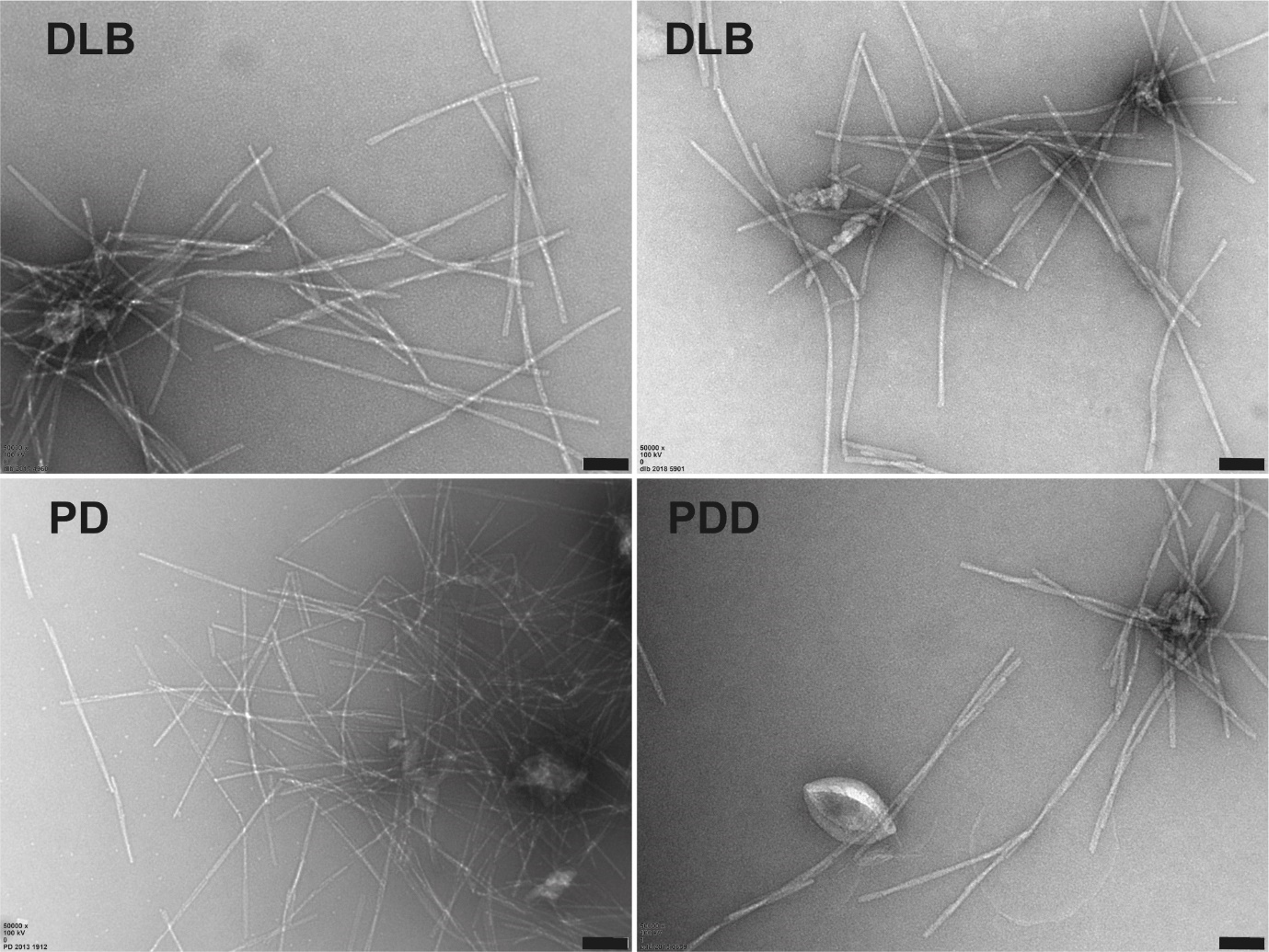


Fig. 11 LBD patient derived EV induce aggregation of wild type alpha-synuclein in RTQuIC assay. Representative TEM images of post-RTQuIC samples from DLB, PDD and PD individuals showing alpha-synuclein fibrils and EV associated with them. Scale bar 100 nm.
